# Toxicological Analysis of Hepatocytes Using FLIM Technique: In Vitro versus Ex Vivo Models

**DOI:** 10.3390/cells10112894

**Published:** 2021-10-26

**Authors:** Svetlana Rodimova, Vadim Elagin, Maria Karabut, Irina Koryakina, Alexander Timin, Vladimir Zagainov, Mikhail Zyuzin, Elena Zagaynova, Daria Kuznetsova

**Affiliations:** 1 Institute of Experimental Oncology and Biomedical Technologies, Privolzhsky Research Medical University, 10/1 Minin and Pozharsky Sq., 603000 Nizhny Novgorod, Russia; elagin.vadim@gmail.com (V.E.); maria.karabut@gmail.com (M.K.); zagainov@gmail.com (V.Z.); ezagaynova@gmail.com (E.Z.); daria.s.kuznetsova@gmail.com (D.K.); 2 Department of Biophysics, N.I. Lobachevsky Nizhny Novgorod National Research State University, 23 Gagarina Ave., 603022 Nizhny Novgorod, Russia; 3 School of Physics and Engineering, ITMO University, 9 Lomonosova St., 191002 St. Petersburg, Russia; irina.koryakina@metalab.ifmo.ru (I.K.); mikhail.zyuzin@metalab.ifmo.ru (M.Z.); 4 Research School of Chemical and Biomedical Engineering, National Research Tomsk Polytechnic University, 30 Lenin Ave., 634034 Tomsk, Russia; a_timin@mail.ru; 5 Institute of Biomedical Systems and Biotechnology, Peter the Great St. Petersburg Polytechnic University, 29 Polytechnicheskaya St., 194064 St. Petersburg, Russia; 6 The Volga District Medical Centre of Federal Medical and Biological Agency, 14 Ilinskaya St., 603000 Nizhny Novgorod, Russia

**Keywords:** FLIM, microfluidic chip, hepatocyte, liver pathology, metabolism

## Abstract

The search for new criteria indicating acute or chronic pathological processes resulting from exposure to toxic agents, testing of drugs for potential hepatotoxicity, and fundamental study of the mechanisms of hepatotoxicity at a molecular level still represents a challenging issue that requires the selection of adequate research models and tools. Microfluidic chips (MFCs) offer a promising in vitro model for express analysis and are easy to implement. However, to obtain comprehensive information, more complex models are needed. A fundamentally new label-free approach for studying liver pathology is fluorescence-lifetime imaging microscopy (FLIM). We obtained FLIM data on both the free and bound forms of NAD(P)H, which is associated with different metabolic pathways. In clinical cases, liver pathology resulting from overdoses is most often as a result of acetaminophen (APAP) or alcohol (ethanol). Therefore, we have studied and compared the metabolic state of hepatocytes in various experimental models of APAP and ethanol hepatotoxicity. We have determined the potential diagnostic criteria including the pathologically altered metabolism of the hepatocytes in the early stages of toxic damage, including pronounced changes in the contribution from the bound form of NAD(P)H. In contrast to the MFCs, the changes in the metabolic state of hepatocytes in the ex vivo models are, to a greater extent, associated with compensatory processes. Thus, MFCs in combination with FLIM can be applied as an effective tool set for the express modeling and diagnosis of hepatotoxicity in clinics.

## 1. Introduction

There are a number of hepatotoxic agents that cause either acute or chronic damage [1]. Among them, ethanol and acetaminophen (APAP) can be considered as two of the most common hepatotoxins. For instance, APAP is a safe analgesic at therapeutic levels. However, an overdose of APAP can cause serious liver injury and even lead to liver dysfunction [2]. High doses of ethanol through alcohol abuse can cause the development of alcoholic liver disease, which can further promote fibrosis, cirrhosis or hepatocellular carcinoma [3].

The metabolism of ethanol and APAP alters the metabolic state of hepatocytes. Importantly, the degree of hepatic toxicity correlates with both the activity of the catalyzing enzyme systems and the availability of glutathione (GSH) in the hepatocytes. An absence of GSH leads to covalent binding of the reactive metabolite N-acetyl-p-benzoquinone imine (NAPQI) to form NAPQI-protein adducts [4,5].

**Figure 1:**
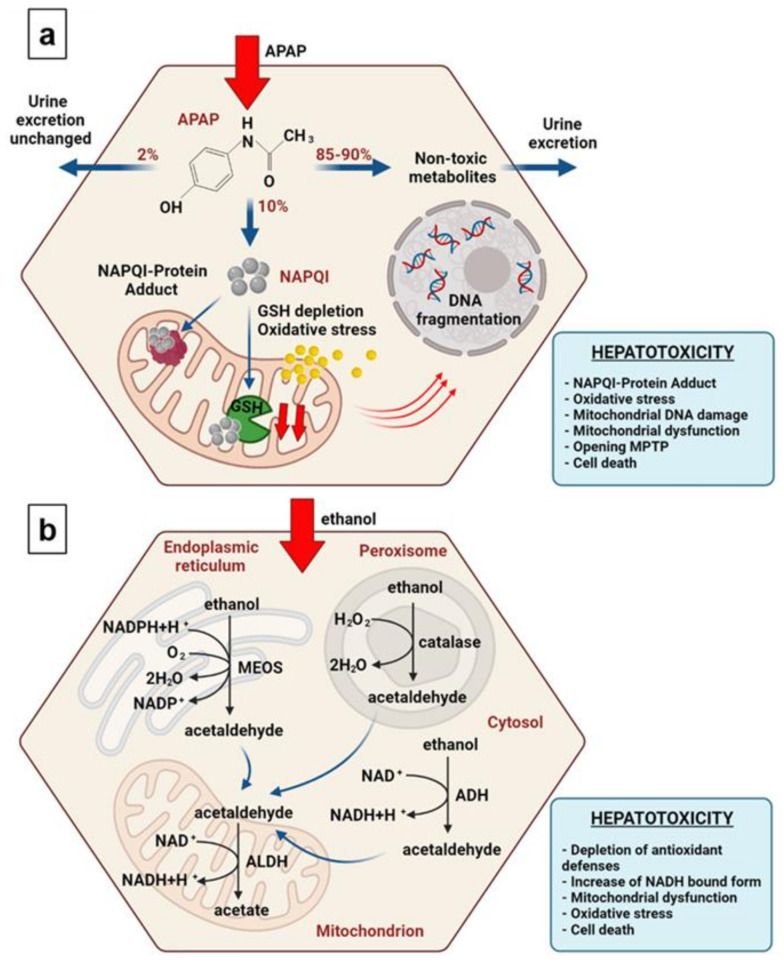
Schematic diagrams of the hepatotoxicity mechanisms. (**a**) APAP and (**b**) ethanol. The percentage distribution of the APAP metabolic pathways is represented by averaged values, and these are in agreement with the published works [6,7].

The mechanisms of hepatotoxicity of APAP and ethanol are illustrated in Figure 1 ((a) is APAP and (b) is ethanol).

APAP: Therapeutic doses of APAP result in the generation of small quantities of NAPQI that are easily detoxified by GSH. Excessive doses of APAP lead to the shunting of more APAP into the cytochrome P450 (CYP) system. Increased metabolism of APAP by CYP, in turn, increases the amount of NAPQI [8,9]. Further, excessive accumulation of NAPQI critically decreases the limited resource of GSH, thereby reducing the detoxifying capacity of the liver. In the absence of free GSH, the excess of NAPQI interacts with the cysteine groups of macromolecules within the hepatocytes, forming NAPQI-protein adducts, leading to mitochondrial dysfunction and the loss of cellular ATP. GSH depletion further contributes to cellular oxidant stress [10,11,12]. Therefore, as final outcomes of the APAP overdose, an alteration in calcium homeostasis, mitochondrial dysfunction with ATP depletion, DNA damage, and intracellular protein modification can occur [13,14,15].

Ethanol: The oxidative pathways of alcohol metabolism involve three enzymes: alcohol dehydrogenase (ADH) in the cytosol; cytochrome P450 (CYP) in the microsomes; and catalase in the peroxisomes [16]. ADH converts alcohol to acetaldehyde and other metabolites. In this reaction, NAD+ is reduced by two electrons to NADH, generating a highly reduced environment in the hepatocyte cytosol. The increased NADH/NAD+ ratio favors hepatic triglyceride accumulation. CYP enzymes (in particular, the microsomal ethanol oxidizing system (MEOS)) that are located mainly in the microsomes and endoplasmic reticulum also contribute to the metabolism of alcohol into acetaldehyde. Acetaldehyde is a highly reactive and toxic byproduct that may contribute to tissue damage because it forms a variety of protein and DNA adducts that promote GSH depletion, lipid peroxidation, and mitochondrial damage [17]. It also contributes to changes in the redox state of the cell and the formation of reactive oxygen species (ROS), [18] which in turn lead to intramitochondrial oxidative stress [19,20,21].

In this regard, decreasing oxidative stress in the hepatocytes than can be induced by the administration of GSH, N-acetylcysteine, and Cyclophilin D can be considered as a common strategy for the treatment of liver diseases caused by APAP or ethanol [15,20,22].

Thus, evaluation of the metabolic state of hepatocytes is an urgent task both for understanding the fundamental mechanisms of hepatotoxicity and for finding new therapeutic agents for liver treatment. Different in vitro, ex vivo and in vivo experimental models can be used for monitoring the level of hepatotoxicity induced by ethanol or APAP.

In general, while rats are commonly used as an animal model for the investigation of ethanol hepatotoxicity, mice are used to study the mechanisms of APAP-induced hepatocyte death. The reason mice were chosen as a suitable model for revealing the influence of APAP on hepatocytes is that rats are resistant to APAP damage. Furthermore, mice are widely used to test APAP hepatotoxicity, since the induced toxic damage in this case is similar to that in humans [22,23]. Although in vivo models reflect the real situation, one important limitation of animal models is that the results can be hard to interpret due to the systemic intoxication induced by the toxic agent. Moreover, in these models, stimulation of chronic damage by a number of toxins (e.g., ethanol) is long-term and can take from 4 to 12 weeks. In addition, the use of animal models presents a wide range of ethical concerns [24,25,26].

Employing ex vivo analysis of tissue slices enables the preservation of the complex multicellular architecture supporting the native components of the intercellular matrix and cellular interactions. However, if it is necessary to analyze metabolic changes directly in the hepatocytes, then the slice model is inferior, because interpreting it is complicated by the influence of the surrounding non-parenchymal cells [27,28].

In vitro models can be more appropriate for simple and fast analysis of hepatotoxicity [29]. In this context, microfluidic technology can help to establish in vitro conditions similar to ex vivo/in vivo environments. Indeed, the microfluidic method opens up the possibility of mimicking the functions of organs in vitro. For instance, with microfluidic chips (MFCs), adjustment of the environmental conditions is possible according to the type of cell. Thus, microfluidics has emerged as a suitable tool for cell manipulation and cell study, as the culture of cells in such flow conditions facilitates the supply of nutrients or other compounds (drugs, toxins) required to study the cellular response [30]. As pointed out in a published study [31], microfluidics can maintain the hepatocytes’ synthetic and metabolic functions. In the same work, microfluidic chips proved to be more sensitive to drug-mediated hepatotoxicity than other methods for most of the drugs tested. Furthermore, in vitro toxicity data have been shown to be positively correlated to in vivo toxicity data. Another successful example of cell lines or primary cells cultured in MFCs is shown in Ref [32], where MFCs were used for the testing of single or multidrug combination chemotherapy at defined doses.

Here, we have systematically studied hepatotoxicity in different research models, including animal models (mice for APAP analysis, and rats for ethanol analysis), precision-cut slices, and an in vitro model using MFCs. All the tested models were compared to each other.

A number of methods are used to analyze the metabolic changes in liver cells caused by toxic damage. In particular, Western blots or enzyme-linked immunosorbent assays provide information about the content of various low molecular weight compounds, as well as specific proteins in the samples [33]. However, these methods do not allow the analysis of freshly prepared samples or living cells, and require tissue destruction and preliminary preparation of the material. Genomic analysis methods have the same drawbacks as they cannot allow monitoring of processes occurring in living cells and do not provide cellular resolution [34]. In addition, although it is possible to analyze blood serum metabolites [35], this method provides only indirect information and is not specific enough. Unlike the above-mentioned methods, fluorescence-lifetime imaging microscopy (FLIM) is label-free, minimally invasive and allows the analysis of fresh tissue samples and living cells [36,37,38,39,40,41]. Currently, very few works have been performed based on analyses of the metabolic state of hepatocytes with toxic damage [42,43]. Furthermore, until now, no comprehensive FLIM analysis has been carried out on the various models, and especially on the MFC model.

A wide range of fluorescence techniques have found broad application in the microscopy of live specimens, since they are extremely sensitive and can be used to study biochemical interactions at the molecular level [44]. However, fluorescence is characterized not only by the spatial distribution of fluorescence intensity and the fluorescence spectrum, but also by the fluorescence lifetime, i.e., the time a fluorescent molecule spends in an excited state before emitting a photon as it decays back to its ground state. The fluorescence lifetime depends on the type of molecule, its conformation, and the way the molecule interacts with its environment [44,45].

Since the metabolic coenzyme NAD(P)H is autofluorescent, it can be monitored nondestructively and without exogenous labels, using optical techniques [46,47,48]. Lakowicz et al. proposed the FLIM technique for distinguishing the free and protein-bound states of the NADH cofactor [49]. Taking this into account, the FLIM approach enables analysis of the metabolic state of various cell types, based on such fluorescence lifetime data and the contributions of the free and bound forms of NAD(P)H to cell metabolism [50,51,52,53,54]. The fluorescence lifetime has both short and long lifetime components that reflect whether the NAD(P)H is in the free or protein-bound state, respectively [46], as it is sensitive to the fluorophore’s microenvironment, thereby providing a method for distinguishing the free and protein-bound components (and their relative contributions).

Cellular energy metabolism is a complex of biochemical pathways that are associated with the synthesis of ATP, so is a sensitive marker of the cellular state and viability. In particular, energy metabolism undergoes changes through the influence of different agents or because of cell damage. Generally, there are two biochemical pathways in a cell responsible for all ATP synthesis. Under normal conditions, most of the ATP in liver cells is produced by the chain of reactions known as the mitochondrial OXPHOS system. It is the bound form of NADH that is involved in this process [55]. In the OXPHOS, NADH acts as the primary donor of protons and electrons for the electron transport chain of the mitochondria. As a result of such NADH oxidation, a proton gradient (electrochemical potential) is formed [56]. This electrochemical potential gradient is required for the production of ATP by ATP-synthetase [57]. The second pathway for ATP synthesis is glycolysis, where a free form of NADH is involved. As a result of this multistage process, two high-energy ATP molecules, two pyruvate molecules, and two water molecules are formed, while two NAD+ molecules are reduced, to form two NADH molecules [58,59]. Under normal conditions, the contribution of glycolysis to energy metabolism is insignificant [60].

However, the pathological effects of toxins (in particular, of ethanol and APAP) can disrupt the functions of mitochondria and the respiratory chain. Such alterations can lead to changes in emphasis of the metabolic pathways for ATP synthesis. Thus, such metabolic changes in hepatocytes can be assessed by the changes in the contributions of the two forms of NADH.

The reduced phosphorylated form, NADPH, is involved in the biosynthesis of fatty acids and steroids, in the pentose phosphate pathway, and in antioxidation defense reactions (GSH metabolism) [61]. Thus, use of the FLIM method allows information to be obtained about the changes in the intensity of these metabolic processes where the different forms of NAD(P)H are involved.

## 2. Materials and Methods

### 2.1. Fabrication of MFCs and Numerical Simulation of Flow in MFCs

The MFCs used in this study consisted of two chambers separated by an array of pillars (an inner chamber and an outer one). The inner chamber contained hydrodynamic traps for the cells. The MFCs were fabricated using a soft lithography method incorporating a silicone elastomer, PDMS. Numerical modeling of the flows in the microchannels of the MFCs was realized with COMSOL Multiphysics 5.6 software. A detailed description of the MFC fabrication procedure and numerical simulation is presented in Supplementary Materials.

### 2.2. Experimental Setup with MFCs

Primary hepatocytes were isolated from the liver of a laboratory animal (mouse/rat) and introduced into the inner chamber of an MFC. Syringe pumps filled with complete cell culture medium (with or without toxins) were connected to the outer chamber of the MFC. The cell culture medium (with or without toxins) was pumped for 3 h or 24 h at 37 °C, 5% CO_2_. The detailed description of the MFC experimental setup is presented in Supplementary Materials.

### 2.3. Animal Model

Acute toxic damage of liver tissue caused by APAP was induced in 10 male mice (C57BL/6). For this purpose, the mice were treated intraperitoneally with doses of 500 mg/kg APAP dissolved in warm saline [62]. Induction of chronic ethanol damage of liver tissue was carried out using 10 male rats (Wistar). For this purpose, we performed daily oral administration of a 40% ethanol solution at a dosage of 3 mL/100 g. A 10% ethanol solution was also added to the animal drinker [63]. The healthy livers of 5 mice and 5 rats were compared as controls.

For the microscopic examination, the whole organ of each animal was isolated, washed with PBS in order to remove the blood, and cut to obtain tissue samples with a size of 0.5 × 0.5 cm.

### 2.4. Precision Cut Liver Slices

In this study, we obtained 15 precision cut liver slices for each experimental group. Fresh hepatic tissue was cut into 1.0 × 1.0 × 0.5 cm^3^ samples. The fixed samples were cut using a stainless steel blade, under buffered conditions with ice-cold PBS. Immediately after cutting, the samples were placed in a 6-well plate with CO_2_-conditioned DMEM supplemented with 10% FBS, 4 mM L-glutamine and antibiotic-antimycotic solution. To induce APAP toxic damage, the slices were then transferred to a 10 mM solution of APAP diluted in DMEM medium with 10% FBS, 4 mM L-glutamine, and antibiotic-antimycotic solution. To induce ethanol toxic damage, further slices were placed in a 25 mM solution of ethanol diluted in DMEM medium with 10% FBS, 4 mM L-glutamine, and antibiotic-antimycotic solution. Slices placed in the medium without toxins were used as a control [28,64]. Further cultivation was carried out in the 12-well plates incubated at 37 °C on an orbital shaker (60 rpm) [65]. After 3 h and 24 h of incubation, slices (from both mice and rats) were visualized using a confocal laser scanning microscope (CLSM).

### 2.5. Multiphoton Microscopy

All the samples were investigated using confocal laser scanning microscopy (CLSM 880) equipped with a Ti:Sapphire femtosecond laser and time-correlated single photon counting (TCSPC). The average laser power used was approximately 10 mW. An oil immersion objective C Plan-Apochromat 40×/1.3) was used to collect the fluorescence signal. From 10 fields of view for each sample, both the NAD(P)H fluorescence intensity images and the FLIM data were acquired. To visualize the NAD(P)H fluorescence, the sample was excited with a wavelength of 750 nm, and the fluorescence signal was detected in the range of 450–490 nm. The FLIM analysis was performed using SPCImage software (Becker & Hickl GmbH, Berlin, Germany) with a biexponential decay model. The following parameters were analyzed in 20–30 regions of the cytoplasm of the cells in each field of view: tm (ps), the amplitude-weighted mean lifetime; t1 (ps), the fluorescence lifetime of the free form of NAD(P)H (the short decay component); t2 (ps), the fluorescence lifetime of the bound form of NAD(P)H (the long decay component); and the relative contributions of the free, a1 (%), and the bound, a2 (%), forms of NAD(P)H. To remove the background signal of the medium in which the hepatocytes were incubated, we used the Threshold option in the SPCImage software. Moreover, the processing was carried out manually, so we could precisely distinguish the areas of cytoplasm in the hepatocytes. A more detailed description of the multiphoton microscopy experiments is presented in Supplementary Materials.

### 2.6. Histology

Histological studies were carried out following a standard procedure that is comprehensively described in Supplementary Materials. Finally, 10 micrographs per sample were acquired using an optical microscope to proceed with a routine morphological analysis.

## 3. Results and Discussion

**Figure 2:**
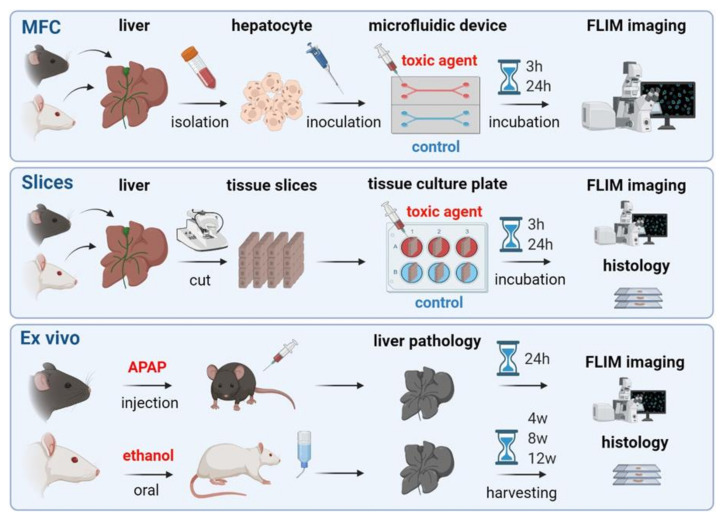
Road map of the steps implemented to study hepatotoxicity using different models. (Microfluidic chip: upper row, slices: middle row, ex vivo: bottom row).

The design of this study is depicted in Figure 2, where the experimental stages of analysis of the hepatocytes’ metabolic state are presented for the MFC model, for precision-cut liver slices, and for the ex vivo samples taken from animals. The main concept of the work was to monitor and compare the metabolic state of the hepatocytes after the introduction of APAP or ethanol, using the FLIM method. The animal model included acute APAP-induced injury caused by a single intraperitoneal injection of the toxin, whereas chronic alcohol injury was created by systematic oral administration of ethanol for 12 weeks. Precision-cut slices, obtained by a microtome, were placed in a culture medium supplemented with APAP or ethanol. In the case of the MFCs, isolated hepatocytes were cultured in a chip, where the relevant toxins (APAP and alcohol) were circulated for a predetermined period of time. In the following sections, we discuss each step in more detail.

### 3.1. MFC Use for Hepatocytes Culture

An MFC was developed to cultivate primary hepatocytes for study of the influence of the toxic effects of APAP and ethanol. The resulting MFC consisted of two chambers: an inner chamber with hydrodynamic traps designed to trap the cells, and an outer chamber (Supplementary Materials, Figure S1). The culture medium was loaded through the outer chamber. To prevent the cells from being removed by the flow of the culture medium, an array of pillars was placed between the outer and inner chambers. Numerical modelling was performed to evaluate the interaction of the culture medium flowing from the outer chamber with the cells in the inner chamber (Supplementary Materials, Figure S2). According to the numerical simulation results obtained, the cells (red dots, d = 70 µm) were captured in the hydrodynamic traps and remained there. Moreover, the captured cells interacted with the flow from the outer chamber when the applied flow rate was in the range from 0.008 µL/s to 0.02 µL/s. Microfluidics attracts a great deal of attention due to the low consumption of materials (both cells and the pumped medium) compared to conventional models such as slices and in vivo (ex vivo) experiments. Furthermore, the use of a microfluidic approach is able to reduce the number of animals used in in vivo testing. In addition, the indisputable advantages of using MFCs are the complete isolation of the cells from the external environment and blood stream imitation. The isolation is achieved by placing the material (liver cells) in the sealed chamber of an MFC. Furthermore, microfluidic systems enable automation of the research process, once the initial loading of the cells into the MFC has taken place. Subsequently, processes such as washing, control of flow rates and reagent concentrations can be fully automated and carried out without the intervention of operators, which therefore reduces the opportunity for error.

### 3.2. Analysis of Metabolic State of Normal Hepatocytes from Mice

At the beginning of the study, the metabolic state of the hepatocytes was evaluated under normal conditions (without adding toxins), using three types of model (in vitro using MFCs, precision-cut slices, and ex vivo). The hepatocytes were imaged using the FLIM technique to reveal aspects of the fluorescence lifetimes: amplitude-weighted mean lifetime (tm), the short t1 (ps) and long t2 (ps) decay components, and the relative contributions of the free (a1, %) and bound (a2, %) forms of NAD(P)H in the hepatocytes for all the models represented. The fluorescence lifetimes of the free and bound forms of NAD(P)H (t1 and t2, respectively) for the MFC model were shorter than those in the slice models and ex vivo samples, at both 3 h and 24 h of cultivation (Supplementary Materials, Figure S12). In a number of earlier works, increased values of t1 and t2 had been shown to be associated with cell hypoxia or cell damage [39,48,66]. It was also reported that a rise in the mean fluorescence lifetime corresponds either to an increase in glycolysis or to a decrease in oxygen tension [67]. High values of t1 and t2 may be associated with a reduced level of synthetic activity in cells. In particular, increased t1 and t2 can be observed in stem cells during their differentiation [68]. We assume that the reason for the shorter fluorescence lifetimes (t1, t2) in the MFC model is due to a decrease in the metabolic activity of the hepatocytes, since they were isolated from tissue. In addition, a slight decline in cell viability may further account for the decrease in fluorescence lifetimes, since the metabolic activity of cells is indirectly related to their viability [39,48,69].

**Figure 3:**
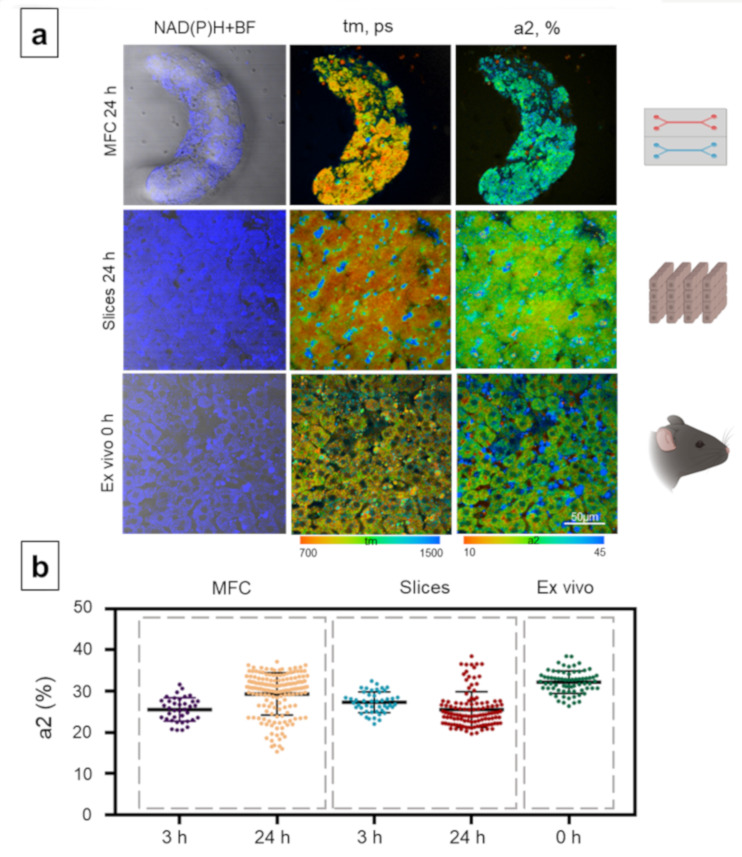
Metabolic imaging of control mouse hepatocytes with the MFC, slice and ex vivo models. (**a**) Combined images of the bright-field and the autofluorescence of NAD(P)H (left row) with pseudocolor-coded fluorescence life-time imaging microscopy (FLIM) images of the NAD(P)H of the hepatocytes (central and right rows), field of view is 213 × 213 μm; (**b**) scatter plots reflecting the distribution of the values of the fluorescence lifetime contributions of the bound form of NAD(P)H. Here, tm (ps) is the amplitude-weighted mean lifetime (tm); a2 (%) is the relative contribution of the bound form of NAD(P)H; scale bar: 50 μm.

The metabolic state of the hepatocytes was analyzed in more detail, based on the fluorescence lifetimes and the relative contributions of the free and bound forms of NAD(P)H, because any change in a1 or a2 reflects a switching of cellular energy metabolism to glycolysis or OXPHOS [70,71]. Since the a2 values are associated with OXPHOS, we subsequently used only this parameter to present the data in the figures. In the case of the MFC model, a statistically significant increase in the contribution of the bound form of NAD(P)H (a2) compared to that at 3 h was observed after 24 h of cultivation (3 h: 25.55% ± 2.8 and 24 h: 29.31% ± 5.1) (Figure 3). In contrast, in the slice model, a slight decline in the contribution of a2 was recorded at 24 h compared to that at 3 h (3 h: 27.26% ± 2.5 and 24 h: 25.54% ± 4.3) (Figure 3). In the ex vivo samples, the contribution of the bound form of NAD(P)H was significantly higher, 32.16% ± 2.7 (Figure 3).

A decrease in the contribution of the NAD(P)H bound form is generally associated with a cellular metabolic shift to a more glycolytic state with a reduced emphasis on OXPHOS. Since OXPHOS is the principal pathway that normal hepatocytes use for obtaining energy, a low a2 value indicates the hepatocytes are suffering damage or hypoxia [72]. Moreover, the lower a2 values found in the MFCs and in the slices compared with the native tissue in the ex vivo samples presumably indicate a reduced OXPHOS level in the hepatocytes in the former due to the disruption of the native tissue structure and partial cell damage inflicted on them during the manipulations. Furthermore, the metabolic switch in hepatocytes to a more glycolytic state is known to be a specific response to their isolation [73].

### 3.3. Analysis of Hepatotoxicity of APAP

Next, the metabolic state of hepatocytes was examined after their exposure to APAP (10 mM solution of APAP) in the three different models. The metabolic activity of the isolated hepatocytes cultured in MFCs was checked after 3 h, since it had previously been demonstrated that the most pronounced metabolic changes under the influence of APAP could be detected in the range of 2–6 h post exposure [74,75]. Analysis of the metabolic state of the hepatocytes after APAP administration revealed that the fluorescence lifetimes t1 and t2 were much shorter in the MFC model compared to those in the slices and ex vivo samples, as had been shown previously, without the addition of APAP (Supplementary Materials, Figure S12). This indicated a decline in the overall metabolic activity of the hepatocytes damaged after exposure to APAP.

**Figure 4:**
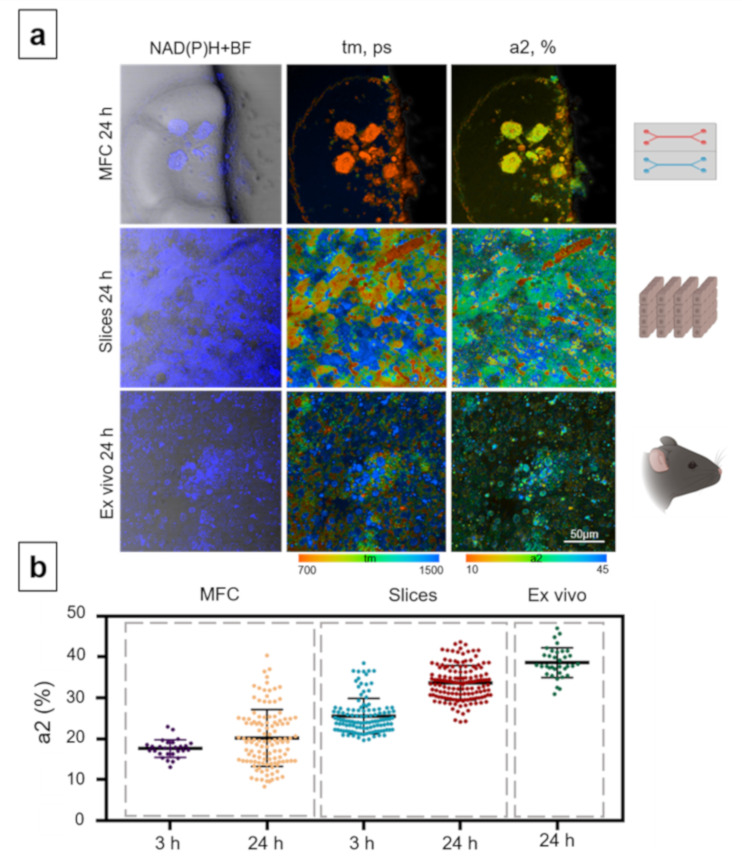
Metabolic imaging of mouse hepatocytes exposed to APAP in the MFC, slice and ex vivo models. (**a**) Combined images of the bright-field and autofluorescence of NAD(P)H (left row) with pseudocolor-coded FLIM images of the NAD(P)H of the hepatocytes (central and right rows), field of view is 213 × 213 μm; (**b**) scatter plots reflecting the distribution of the values of the fluorescence lifetime contributions of the bound form of NAD(P)H. Here, tm (ps) is the amplitude-weighted mean lifetime (tm); a2 (%) is the relative contribution of the bound form of NAD(P)H; scale bar: 50 μm.

On analyzing the contribution of bound NAD(P)H, a2 (%), the MFC model showed a reduced contribution of the bound form of NAD(P)H compared to hepatocytes without the addition of APAP (3 h: 17.6% ± 2.1, and 24 h: 20.16% ± 6.9) (Figure 4).

As shown in our previous work, OXPHOS predominates in normal hepatocytes and, accordingly, the contribution of the bound form of NAD(P)H is high [53]. The drop in the contribution of the bound form of NAD(P)H in isolated hepatocytes is consistent with the known mechanisms of APAP hepatotoxicity. Specifically, APAP overdose leads to mitochondrial dysfunction and the disruption of the mitochondrial respiratory chain [12]. Moreover, NAPQI inhibits both NADH (complex I)- and succinate (complex II)-driven respiration in isolated mouse liver mitochondria or permeabilized mouse hepatocytes, apparently without affecting the complexes III and IV [76]. A decrease in the contribution of the bound form of NAD(P)H is also associated with a drop in the ATP content in hepatocytes, which has previously been reported [10,11]. In Figure 4b, a large standard deviation of a2 in the MCF model was observed, which can be attributed to significant differences in the metabolic states of the hepatocytes isolated from tissue. In this state, hepatocytes have a varying baseline level of metabolic activity. In addition, this may be due to different individual sensitivity of cells to the toxin. Models where the tissue structure is conserved (ex vivo and slices) are better able to maintain cell homeostasis, and, therefore, the metabolic state is more stable.

In the slice model, there was a sharp increase in the value of the contribution of a2 after 24 h of cultivation (3 h: 29.42% ± 3.5, and 24 h: 33.66% ± 4.2) (Figure 4). A similar trend was observed in the ex vivo model; there was a significant growth in the values of the a2 contribution at 24 h after the introduction of APAP (38.59% ± 3.6) (Figure 4). Both in the slice model and in the animal model, the values of a2 significantly increased compared to the control data (no added toxin). Thus, in the slices and ex vivo samples, the characteristic decrease in the intensity of OXPHOS that was observed in the MFC model, was not present. The low a2 values in the case of the MFC model can be explained by the enhanced sensitivity of isolated hepatocytes to the toxins. This finding is in agreement with previous studies. For instance, it had been demonstrated that 24 h after the exposure of isolated hepatocytes to APAP, a more rapid decrease in the levels of ATP and GSH occurred, indicating a larger decrease in hepatocyte viability [77]. By contrast, it is assumed that, where the tissue structure is maintained (in the slices and ex vivo samples), when the action of APAP is stopped, the mechanisms of ATP resynthesis are activated, this also explaining their increase in the contribution of the bound form of NAD(P)H [77,78].

Based on the above results, we can conclude that the isolated hepatocytes in the in vitro model (in the MFC model) are more sensitive to the influence of the toxin (APAP). Correspondingly, the preserved tissue structure (of the slice and ex vivo models) allows a better maintenance of hepatocyte homeostasis and provides for compensatory processes when exposed to APAP.

### 3.4. Analysis of the Metabolic State of Normal Hepatocytes from Rats

The metabolic state of hepatocytes from rats was analyzed. As previously mentioned, three types of models (MFC, slices and ex vivo) were used. The fluorescence lifetimes (t1 and t2, ps) of the free and bound forms of NAD(P)H were similar in all three models (Supplementary Materials, Figure S13). This result indicates the stability of the metabolic activity of the hepatocytes in all the tested models. Thus, isolated rat hepatocytes were shown to have a more stable metabolic state compared to the isolated mouse hepatocytes (which showed changed values of t1 and t2).

**Figure 5:**
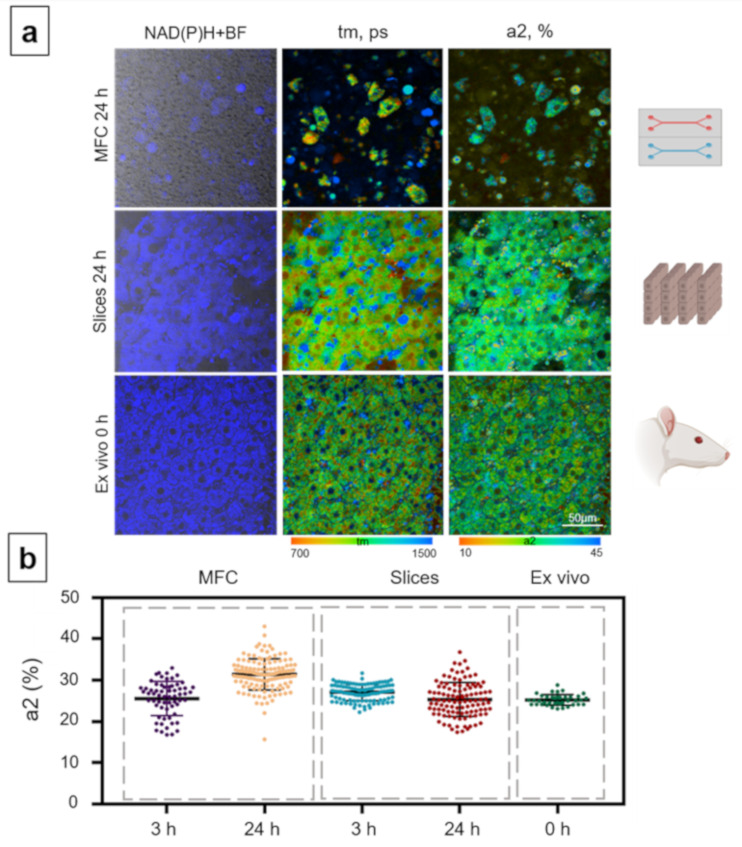
Metabolic imaging of control rat hepatocytes for the MFC, slice and ex vivo models. (**a**) Combined images of the bright-field and autofluorescence of NAD(P)H (left row) with pseudocolor-coded FLIM images of the NAD(P)H of the hepatocytes (central and right rows), field of view is 213 × 213 μm; (**b**) scatter plots reflecting the distribution of the values of the fluorescence lifetime contributions of the bound form of NAD(P)H. Here, tm (ps) is the amplitude-weighted mean lifetime (tm); a2 (%) is the relative contribution of the bound form of NAD(P)H; scale bar: 50 μm.

The in vitro MFC model with normal (untreated) rat hepatocytes showed a significant rise in the contribution of the bound form of NAD(P)H after 24 h of cultivation, compared with 3 h (3 h: 25.54% ± 4.1, and 24 h: 33.65% ± 2.7) (Figure 5). For both rats and mice, lower a2 values for the MFC model were observed after 3 h, compared to 24 h. This result can be explained by the stabilization of the metabolic state of the hepatocytes within 24 h after isolation from the tissue. It is known that oxidative phosphorylation predominates in normal hepatocytes [79]. Thus, we can conclude that within 24 h there is a shift in the metabolic activity of the hepatocytes to the specific level. The slice model showed a slight decrease in the contribution of a2, but in general, the metabolic state of hepatocytes in this model was stable (3 h: 27.05% ± 4.4, and 24 h: 25.33% ± 2.3) (Figure 5). In the hepatocytes of the ex vivo samples, the contribution of a2 was 25.2% ± 1.3 (Figure 5). Thus, the metabolic state of the hepatocytes in all three models was similar after 24 h, following the initial temporary shift in metabolic state of hepatocytes in the MFCs.

### 3.5. Analysis of the Hepatotoxicity of Ethanol

Next, we studied metabolic changes in the hepatocytes cultured in MFCs, as well as in hepatocytes present in the slices and in ex vivo samples under exposure to ethanol. Modeling chronic liver injury under the influence of ethanol usually takes 4–12 weeks [25,26]. In our work, the induction of chronic ethanol hepatotoxicity was carried out for 12 weeks. Based on previous observations, the values of the fluorescence lifetimes of the free (t1) and bound (t2) forms of NAD(P)H in the MFC model and in the slices were similar. The values of t1 and t2 for the ex vivo samples were significantly higher in comparison with the other models (Supplementary Materials, Figure S13). It is known that t2 is composed of the signal from the bound form of NADH and NADPH (the phosphorylated form of NADH). However, in most cases, the contribution of NADPH is negligible and can be neglected [80]. The standard values of t2 in the cells ranged from 1500 to 2200 ps. The unchanged lifetimes of the bound form of NAD(P)H in the MFC and slice models indicated that NADPH is not involved in the hepatocyte metabolism (similar to the case of APAP hepatotoxicity). The possible reason for this is the reduced content of GSH, since in these models (MFC and slices), the possibilities of compensatory processes are reduced. As previously reported, chronic ethanol consumption selectively decreases the mitochondrial GSH level, presumably because of the impaired uptake of GSH from the cytosol to the mitochondrial matrix [81].

However, in the case of the ex vivo samples, there was an increase in the values of t2 indicating an increase in the emphasis on the processes associated with NADPH. Thus, the obtained data can be explained by the involvement of antioxidant defense processes with an increase in GSH synthesis under the influence of ethanol [63,82,83,84].

**Figure 6:**
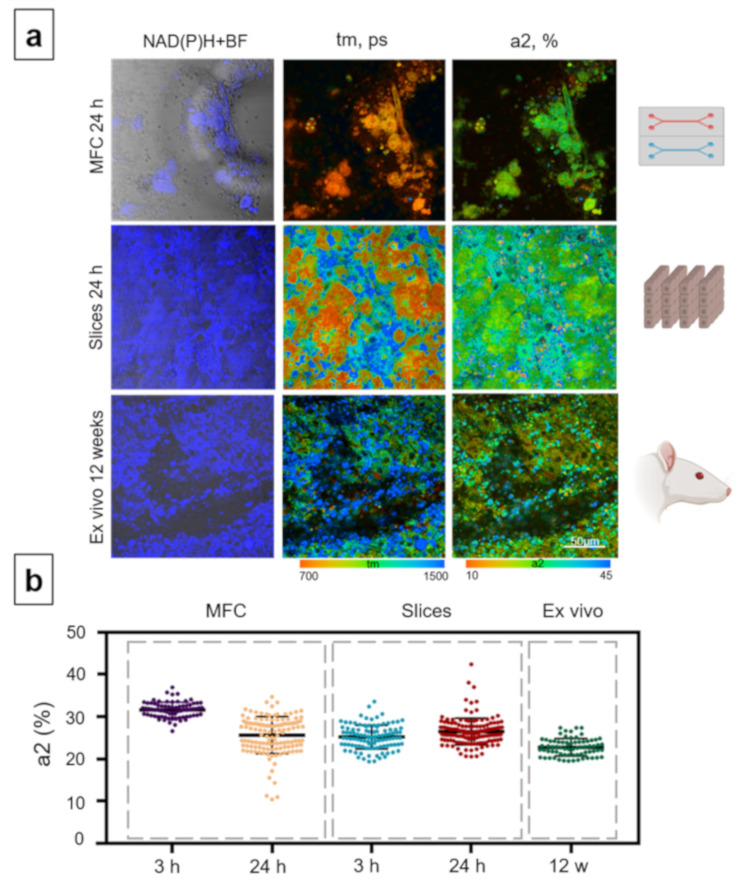
Metabolic imaging of rat hepatocytes exposed to ethanol in the MFC, slice and ex vivo models. (**a**) Combined images of the bright-field and autofluorescence of NAD(P)H (left row) with pseudocolor-coded FLIM images of the NAD(P)H of the hepatocytes (central and right rows), field of view is 213 × 213 μm; (**b**) scatter plots reflecting the distribution of the values of the fluorescence lifetime contributions of the bound form of NAD(P)H. Here, tm (ps) is the amplitude-weighted mean lifetime (tm); a2 (%) is the relative contribution of the bound form of NAD(P)H; scale bar: 50 μm.

In the MFC model, it was shown that the contribution of a2 was significantly lower after 24 h of rat hepatocyte cultivation compared to 3 h (3 h: 31.62% ± 1.8; 24 h: 24.44% ± 4.9) (Figure 6). In the microfluidic approach, the a2 values after 24 h of culturing were lower than in the normal hepatocytes. In the animal model (ex vivo), the value of the contribution of a2 after 12 weeks of induction of chronic pathology was 22.75% ± 2 (Figure 6); this value is also lower than in the liver tissue of healthy animals.

The results obtained in the animal model and in the isolated hepatocytes are in good agreement with the previous studies. In the rat model after chronic ethanol exposure, a profound defect in mitochondrial metabolism was reported [81], resulting in depressed mitochondrial protein synthesis and the associated loss of complexes involved in the mitochondrial electron transport chain [85,86,87]. These chronic ethanol-induced alterations can also lead to depressed respiratory capacity and impaired OXPHOS, which are critical in the development of alcoholic liver injury [88,89].

Several studies have demonstrated a decrease in ATP content when exposed to ethanol [90,91]. Indeed, mitochondria isolated from the liver of alcohol-fed animals contain lower amounts of respiratory chain components compared to the mitochondria of non-treated animals. In addition, it has been shown that animals exposed to alcohol had lower levels of the enzyme complex that mediates ATP production. As a result, the rate of ATP synthesis in such liver mitochondria is depressed as well, leading to an overall decline in ATP content in the hepatocytes [92]. Therefore, disruption of the mitochondrial respiratory chain leads to the decreased contribution of the bound form of NAD(P)H seen in the ex vivo models and in isolated hepatocytes.

By contrast, according to the data obtained, the metabolic state remained approximately constant between 3 h and 24 h (3 h: 25.18 ± 3.9, and 24 h: 26.46 ± 2.9) in the slice model (Figure 6). The exposure to ethanol also disrupted the processes of glycolysis, additionally contributing to a decrease in the ATP content in the cells and to their further damage. However, compensatory processes often occur in liver cells at the early stages of ethanol exposure [90,91,92]. Thus, the stability of the a2 value in the case of the slice model may be associated with a simultaneous decrease in two processes at once (glycolysis and OXPHOS) and compensatory processes occurring as a result of the slice model being more complex compared to the isolated cells, allowing the cells in the slices to maintain the balance of their metabolic state.

It can be concluded that the results obtained in the models of isolated hepatocytes and in the animal model are consistent with each other and reflect the known mechanisms of ethanol hepatotoxicity more accurately than do the slices. The exposure of slices to ethanol for 24 h is not enough to allow for the analysis of the metabolic changes that had been clearly observed in the slices in the APAP hepatotoxicity model. This is probably associated with the resistivity of the hepatocytes to the toxic effects of ethanol in the slice model due to the preservation of the native tissue structure [77,78].

## 4. Histological Analysis of Hepatocytes

**Figure 7:**
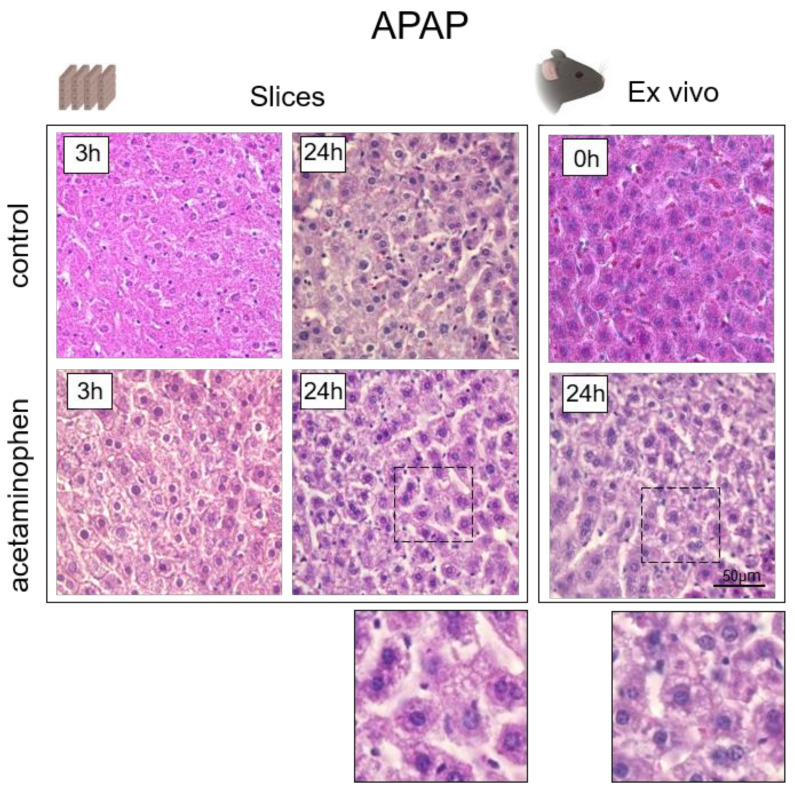
Histological images of liver tissue in the normal state and when exposed to APAP. Hematoxylin and eosin staining, ×400; scale bar: 50 μm.

Well-established histological alterations of hepatocytes after APAP administration include extensive centrilobular coagulative necrosis of the hepatocytes, the dilatation of sinusoids, and microvesicular and macrovesicular steatosis. The microvesicular steatosis occurs as a consequence of direct toxicity on the mitochondria and their oxidative processes. Macrovesicular steatosis corresponds to triglyceride accumulation due to defects in lipoprotein metabolism, damage to the plasma membrane, or to increased lipid delivery to the hepatocytes following an increased synthesis or mobilization [93]. In our case, histological analysis confirmed the presence of similar pathological changes associated with exposure to APAP, both in the slices and in the ex vivo samples obtained from animals. We observed severe fatty infiltration, dilatation of sinusoids and cell edema. However, the number of necrotic hepatocytes was insignificant (Figure 7).

**Figure 8:**
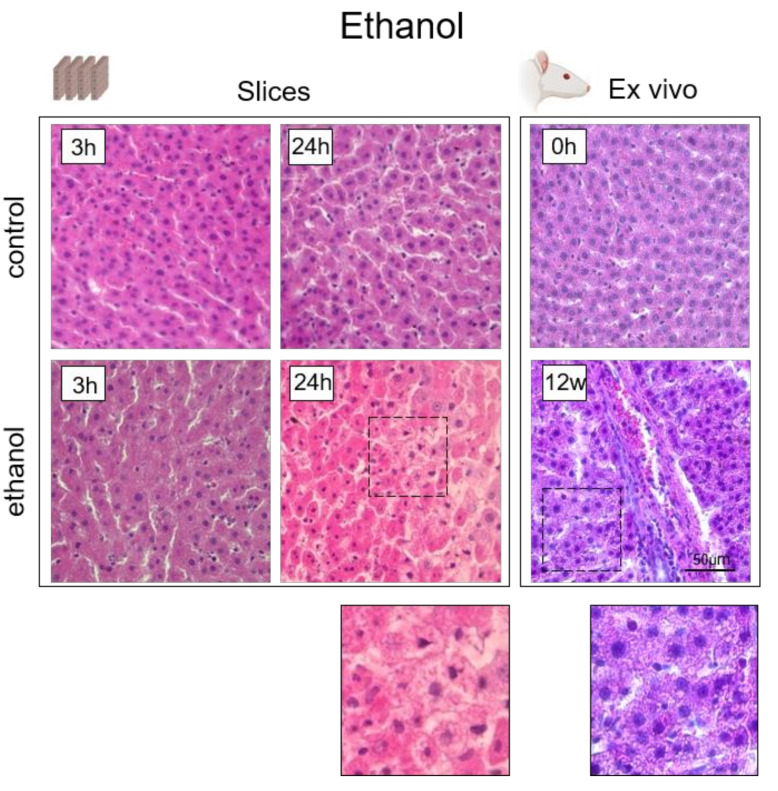
Histological images of liver tissue in its normal state and when exposed to ethanol. Hematoxylin and eosin staining, ×400; scale bar: 50 μm.

Under exposure to ethanol, histological changes include coagulation necrosis of the hepatocytes, swollen hepatocytes, and cytoplasmic vacuolation (vesicular steatosis). With chronic exposure, serious alterations of the normal parenchyma architecture and fibrosis occur [94]. Here, we have also confirmed the presence of pathological changes associated with exposure to ethanol. In the slices, we revealed necrosis of the hepatocytes and a pronounced fatty infiltration of the cells. As the process of fibrosis is complex and systemic, its development cannot be observed in the slice model [95]. However, in the ex vivo model, in addition to fatty infiltration, we did observe initial signs of tissue fibrosis (Figure 8).

Thus, toxic damage induced in liver tissue was confirmed for both toxins. The metabolic state of the hepatocytes in the MFC model corresponded to the known mechanisms of toxic damage, indicating the relevance and potential value of this model.

## 5. Conclusions

The comparative study on the hepatotoxicity of APAP and ethanol was performed using three models: MFCs, tissue slices, and an animal model (ex vivo samples). In the presence of APAP (mouse ex vivo model), we showed that conservation of the tissue structure (in the slices and ex vivo models) allows a better maintenance of the metabolic state of the hepatocytes and enables compensatory processes to occur, while in the MFC model, we revealed a characteristic decrease in the intensity of OXPHOS and an increased sensitivity of the isolated hepatocytes to the toxin. In the presence of ethanol (rat model), the values of t1 and t2 for the ex vivo samples were significantly higher in comparison to the other models, probably due to the higher contribution of NADPH, as a compensatory reaction. In the MFC and ex vivo models, we showed that the emphasis on OXPHOS was significantly reduced, which precisely reflects the known mechanisms of ethanol hepatotoxicity. At the same time, no significant changes in a2 were revealed in the slice model; thus, the exposure of slices to ethanol for 24 h is apparently not enough to develop detectable metabolic changes in the hepatocytes. This was similar to the APAP hepatotoxicity model, where no metabolic changes were observed, either. In general, the changes in the metabolic state of isolated hepatocytes in the MFC model corresponded to the known mechanisms of hepatotoxicity of APAP and ethanol, and we assume that such MFCs can therefore be used as effective tools for express modeling of the hepatotoxicity of various agents. The FLIM technique, which was used to analyze the metabolic state of the hepatocytes, has demonstrated its efficacy for the express assessment of the effects of various toxic agents and can be further successfully translated into clinical settings.

## Data Availability

All relevant data are within the paper and its Supplementary Materials.

## Supplementary Materials

The following are available online at https://www.mdpi.com/article/10.3390/cells10112894/s1, Supplementary Materials (presented in PDF) includes extended information on the using materials, fabrication of microfluidic chips, numerical simulation of flows in MFCs, MFCs experimental setup, animal modeling, precision cut liver slices preparation technique, multiphoton microscopy setup, FLIM data on fluorescence lifetimes values of NAD(P)H (t1 and t2, ps), and histological analysis protocol.
