# Supplementary material for: Toxicological Analysis of Hepatocytes Using FLIM Technique: In Vitro versus Ex Vivo Models
Source: Cells. 2021 Oct 26;10(11):2894. doi: 10.3390/cells10112894 (PMC8616382; doi:10.3390/cells10112894)
Supplement: Supplementary file 1 [file cells-10-02894-s001.zip › cells-1383295-supplementary.pdf]

Additional information

Toxicological analysis of hepatocytes using FLIM technique: *in vitro* versus *ex vivo* models

Svetlana A. Rodimova[1,2], Vadim V. Elagin[1], Maria M. Karabut[1], Irina G. Koryakina[3], Alexander S. Timin[4,5], Vladimir E. Zagainov[1,6], Mikhail V. Zyuzin[3], Elena V. Zagaynova[1,2], Daria S. Kuznetsova[1,2*]

[1]Institute of Experimental Oncology and Biomedical Technologies, Privolzhsky research medical university, 10/1 Minin and Pozharsky sq., Nizhny Novgorod, Russia
[2]N.I. Lobachevsky Nizhny Novgorod National Research State University, 23 Gagarina ave., Nizhny Novgorod, Russia
[3]School of Physics and Engineering, ITMO University, 9 Lomonosova st., St. Petersburg, Russia
[4]Research School of Chemical and Biomedical Engineering, National Research Tomsk Polytechnic University, 30 Lenin ave., Tomsk , Russia
[5]Peter the Great St. Petersburg Polytechnic University, 29 Polytechnicheskaya st., St. Petersburg, Russia
[6]The Volga District Medical Centre of Federal Medical and Biological Agency, 14 Ilinskaya st., Nizhny Novgorod, Russia

*corresponding author: srodimova123@gmail.com

Table of contents

1. Materials 1
2. Fabrication of MFCs 2
3. Numerical simulation of flows in MFCs 2
4. MFCs experimental setup 3
5. Animal model 4
6. Precision Cut Liver Slices 5
7. Multiphoton microscopy 5
8. Histological analysis 15
References 15

1. Materials

For the MFC fabrication: polydimethylsiloxane (PDMS, SYLGARD™ 184 Silicone Elastomer kit, Dow Corning, USA), which consists of two parts: silicone elastomer base (PDMS base) and curing agent. Glass substrate (Minilab, Russia).

For hepatocyte isolation and culture: Zoletil 100 (Virbac, France), Phosphate buffered saline (PBS) (PanEco Ltd., Russia), Accutase™ (Gibco, USA), DMEM (PanEco Ltd., Russia), FBS (Gibco, USA), Antibiotic-Antimycotic (100X) DMEM/F-12, GlutaMAX™ (Gibco, USA), L-glutamine (PanEco Ltd., Russia), dexamethasone (STEMCELL Technologies, USA), and trypan blue (PanEco Ltd., Russia).

For histological analysis: Eosin solutions, Ethanol and Isopropyl, and Hematoxylin solution (Mayer's) were purchased from BioVitrum (Russia).

2. Fabrication of MFCs

The developed MFC consisted of two chambers: an inner chamber with hydrodynamic traps and an outer chamber. The geometrical parameters of the inner chamber are as follows: 17 mm (length) x 2.8 mm (width) x 0.1 mm (height). The parameters of the outer channel are as follows: 27 mm (length) x 5.2 mm (width) x 0.1 mm (height). An array of 108 hydrodynamic traps with the following parameters: 0.07 mm (inner diameter) x 0.1 mm (outer diameter) is placed in the inner channel (Kukhtevich et al. 2015). Before the traps, 4 guiding lines are placed to enable an even distribution of the input flow. The chambers are separated by an array of pillars (Figure S1a).

The mold was made by etching the silicon substrate (depth of etching was 100 μm). MFCs were further fabricated by "soft lithography" technique using silicone elastomer PDMS. PDMS mixture of 1:10 ratio of curing agent to polymer base was obtained and poured onto a silicon mold. Then the uncured PDMS was degassed in a vacuum chamber for 20 min and cured at 80 °C during 2 h. Afterwards, the PDMS replica was sealed to a cover glass (170 μm) by oxygen plasma treatment. The topology and the resulting MFC are presented in Figure S1b.

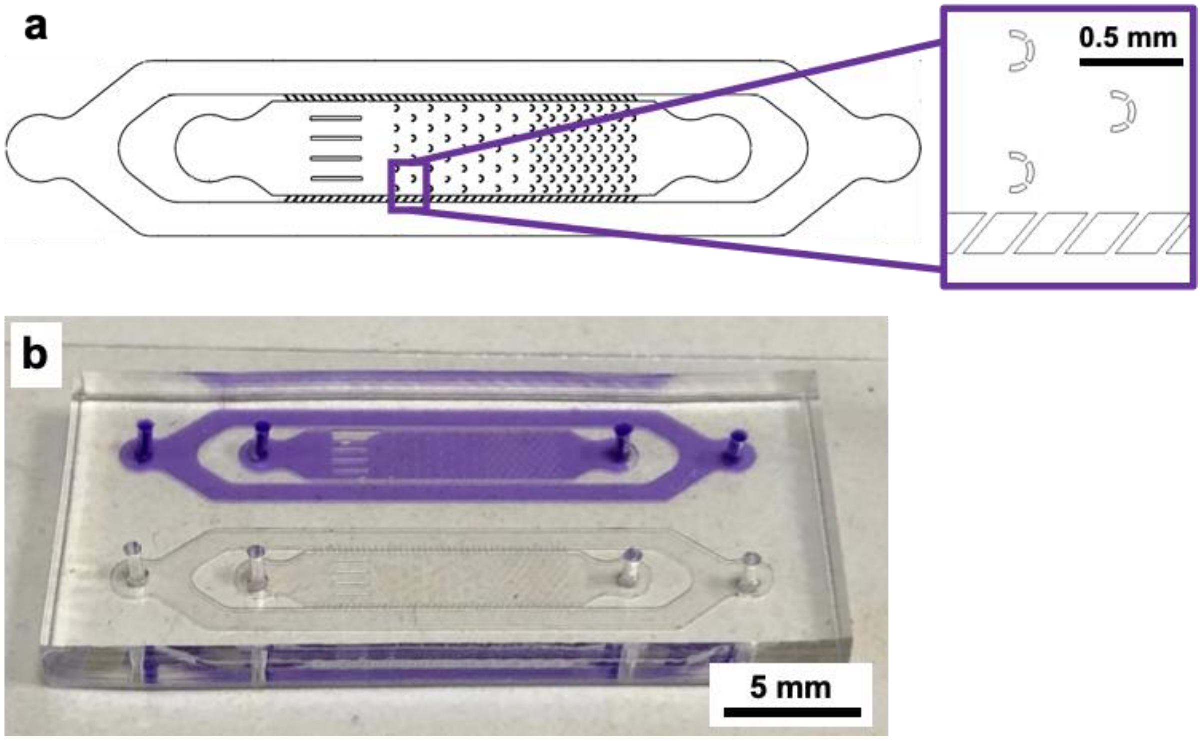

Figure S1. MFC designed for the study. (a) - topology design (inset: view of the hydrodynamic traps for the cells), (b) - digital image of an MFC

3. Numerical simulation of flows in MFCs

The COMSOL Multiphysics 5.6 software was used for the numerical modeling of flow propagation processes through MFC channels by the finite element method. A two dimensional model with *Laminar flow* and *Particle tracing for Fluid flow* physics were used.

The numerical simulation was performed for the flow rate Q = 0.02 μL/s considering the height of the channel equal h = 100 μm. Carrying fluid was considered as a water solution with density **ρ** = 997 kg/m^3 and dynamic viscosity μ = 8.9e-4 Pa s. Particles diameter was taken as d = 70 μm.

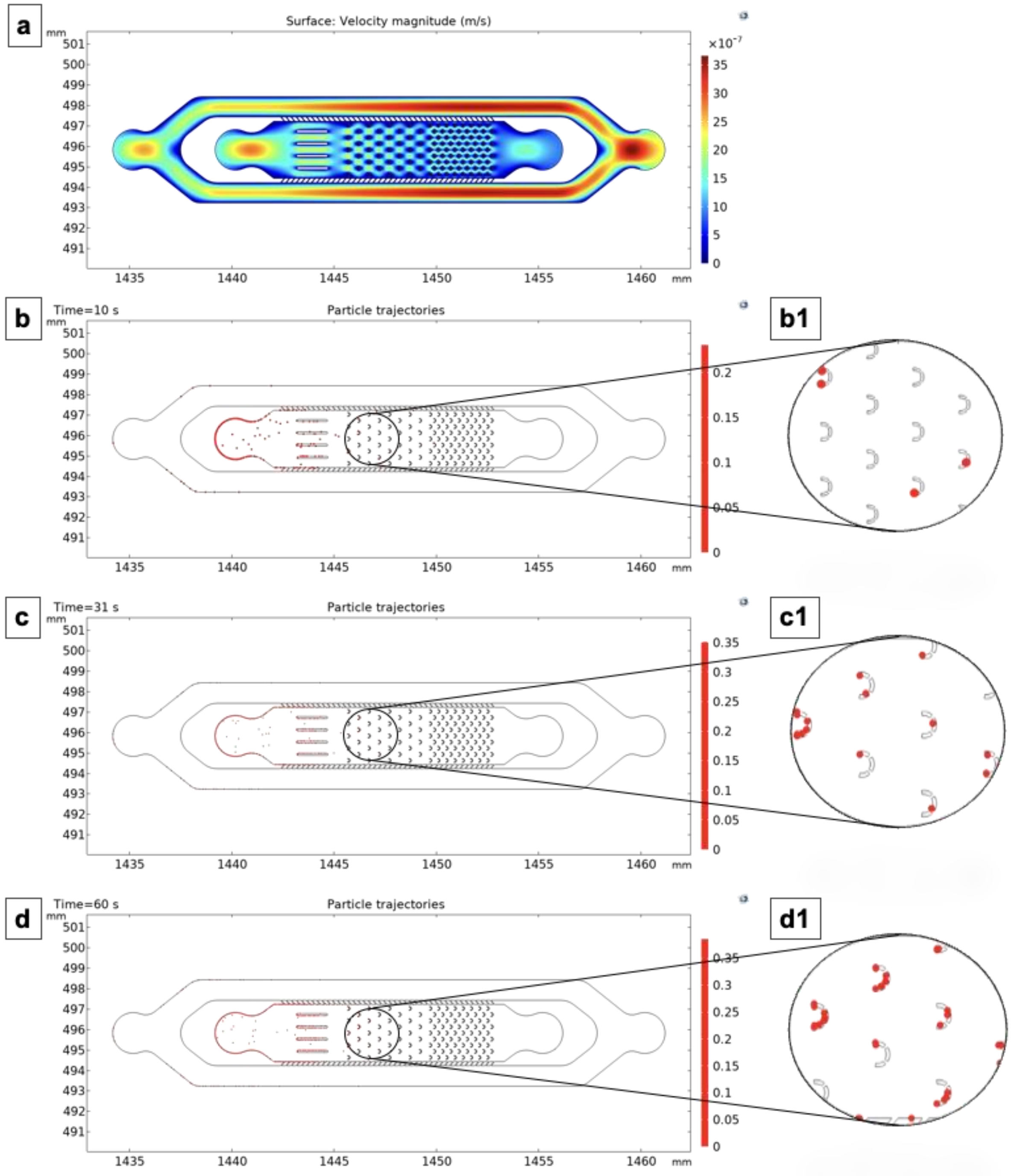

Figure S2. Numerical modeling of the flow distribution in the channels of an MFC. (a) Velocity distribution in an MFC (flow rate 0.02 μL/s); (b) cells' trajectories at time t = 10 s; b1. A magnified area of the cell behavior simulation near the hydrodynamic traps at t = 10 s; (c) cell trajectories at time t = 31 s; c1. A magnified area of the cell behavior simulation near the hydrodynamic traps at t = 31 s; (d) cell trajectories at t = 60 s; d1. A magnified area of the cell behavior simulation near the hydrodynamic traps at t = 60 s

4. MFCs experimental setup

Primary hepatocytes were used for culturing in MFCs. To isolate the hepatocytes, liver samples were obtained after the entire organ was removed from a laboratory animal and washed with saline to remove residual blood. For this, animals were anesthetized with Zoletil at a concentration of 80 mg/kg by intramuscular injection. Then, the obtained liver was

cut into pieces 1-3 mm in size, washed three times with PBS, and then placed in the enzyme Accutase™ (Gibco, USA) to remove the extracellular matrix and incubated for 2 h in an orbital shaker (150 rpm) at 37 °C. The enzyme Accutase was chosen because of its gentle effect on cells, as it does not damage the surface factors of hepatocytes. This aspect is important, since there is evidence that the loss of a surface factor during collagenase preparation of isolated hepatocytes can be responsible for the early and conspicuous phenotypic changes (Guillouzo 1998). Then, the cell suspension was washed to remove the tissue debris by centrifugation at 200xg for 3 min. The collected cells were washed three times in DMEM medium (PanEco Ltd., Russia), 4 mM L-glutamine (PanEco Ltd., Russia) with 10% FBS (Gibco, USA), 0.1 μM dexamethasone (STEMCELL Technologies, USA) and antibiotic-antimycotic consisting of 100 unit/mL of penicillin, 100 μg/mL of streptomycin and 25 μg/mL of Fungizone™ (Gibco, USA). At this stage, cells were counted, and the cell viability was assessed using trypan blue (PanEco Ltd., Russia) (Guillouzo 1998; LeCluyse et al. 2005; Li WC et al. 2010), which showed 80-90% viable hepatocytes (data not shown).

The design of the experimental setup with MFCs is depicted in Figure S3.

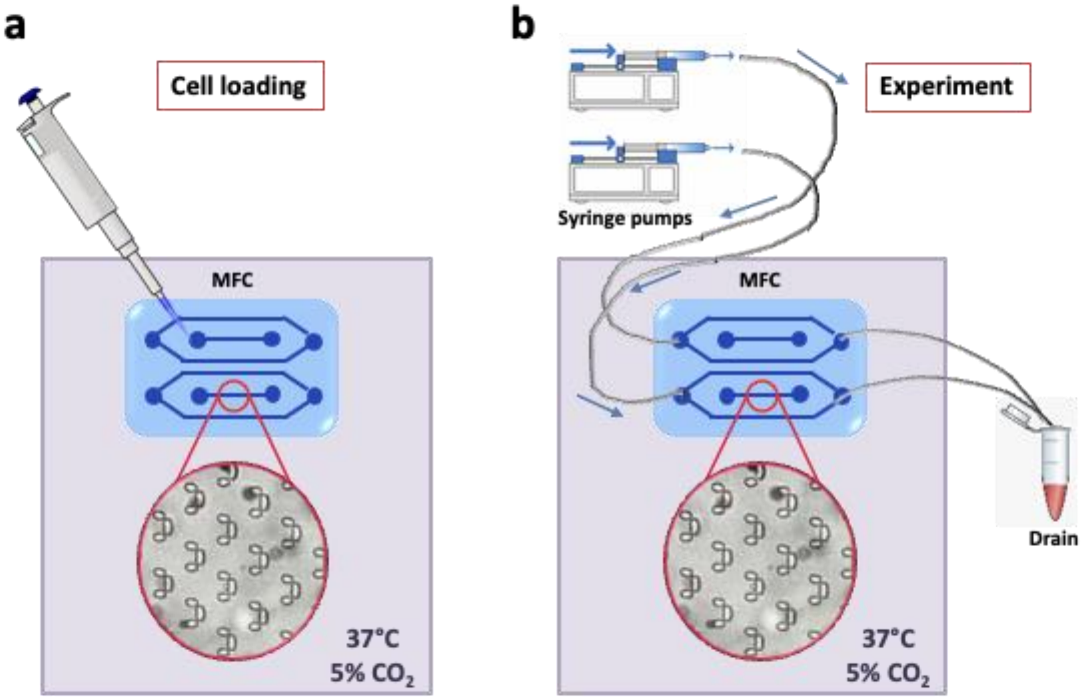

Figure S3. Scheme of the experimental setup. (a) Loading of the cells into the inner chamber of MFC; (b) microfluidic experimental setup for toxicity testing

Both the inner and the outer chambers of an MFC were filled with complete cell culture medium using a mechanical pipette. Then capillaries with attached 2 mL syringes were filled with culture medium with or without toxic agent (10 mM solution of APAP diluted in DMEM, or 25 mM solution of ethanol diluted in DMEM) and connected to the outer chamber of an MFC. The cells were carefully loaded by a mechanical pipette (Gilson, USA) into the inner chamber. The syringes were placed in pumps that provide a medium flow rate of 0.02 μL/s. The whole system was kept at 37 °C, 5% CO_2. The syringe with a toxic agent was replaced by another one with fresh culture medium after 3 h and left for another 21 h (Figure S3).

5. **Animal model**

Acute toxic damage of liver tissue caused by APAP was induced using 10 males of mice (C57BL/6). For this purpose, the mice were treated intraperitoneally with doses of 500 mg/kg APAP dissolved in a warm saline (Li et al. 2010). Induction of chronic ethanol damage of liver tissue was carried out using 10 males of rats (Wistar). For this purpose, we performed daily oral administration of 40% ethanol solution at a concentration of 3 mL/100 g. A 10% ethanol solution was also added to the animal drinker (Hall et al. 2001). Healthy livers of 5 mice and 5 rats were examined as a control.

For the microscopic examination, the whole organ of an animal was isolated, washed with PBS in order to remove blood and cut to obtain tissue samples with the size of 0.5 x 0.5 cm.

6. Precision Cut Liver Slices

Fresh hepatic tissue was cut into 1.0 × 1.0 × 0.5 cm^3 samples. For this, a 7000 smz-2 vibrating microtome (Campden Instruments Ltd., UK) was used to obtain tissue slices. The samples were fixed with a 3 uL of methyl cyanoacrylate glue dropped onto specimen plates for 5 s. Afterwards, the fixed samples were cut using a stainless steel blade (7550-1-ss, Campden Instruments Ltd., UK), under buffered conditions with ice-cold PBS. The following slicing settings were used: the frequency was 80 Hz, the oscillation amplitude was 2 mm, the sectioning speed was 0.4 mm/s, and the step size was 500 µm. Immediately after cutting, samples were placed in a 6-well plate with CO_2-conditioned DMEM (PanEco Ltd., Russia) supplemented with 10% FBS (Gibco, USA), 4 mM L-glutamine (PanEco Ltd., Russia), and antibiotic-antimycotic consisting of 100 unit/mL of penicillin, 100 µg/mL of streptomycin and 25 µg/mL of Fungizone™ (Gibco, USA). To induce APAP toxic damage, the obtained slices were placed in a 10 mM solution of APAP diluted in a DMEM medium with 10% FBS, 4 mM L-glutamine (PanEco Ltd., Russia), and antibiotic-antimycotic solution consisting of 100 unit/mL of penicillin, 100 µg/mL of streptomycin and 25 µg/mL of Fungizone™ (Gibco, USA). To induce ethanol toxic damage, the slices were placed in a 25 mM solution of ethanol diluted in DMEM medium with 10% FBS, 4 mM L-glutamine, and antibiotic-antimycotic solution. A slice placed in the medium without toxins was used as a control (Jaeschke et al. 2011; Palma et al. 2019). Further cultivation was carried out in 12-well plates and incubated at 37 °C on an orbital shaker (60 rpm) (Van Midwoud et al. 2011). After 3 and 24 h of incubation, slices (both from mice and rats) were visualized using a confocal laser scanning microscope (CLSM).

7. Multiphoton microscopy

Investigation of all the samples was performed using a CLSM 880 (Carl Zeiss, Germany) equipped with a Ti:Sapphire femtosecond laser (repetition rate: 80 MHz, pulse duration less than 100 fs) and a time-correlated single photon counting (TCSPC) system (Simple-Tau 152, Becker & Hickl GmbH, Germany). The average used laser power was about 10 mW. An oil immersion objective C Plan-Apochromat 40x/1.3 was used to collect the fluorescence signal. From 10 fields of view for each sample, both the NAD(P)H fluorescence intensity images and the FLIM data were acquired. To visualize the NAD(P)H fluorescence, the sample was excited with a wavelength of 750 nm, and the fluorescence signal was collected in the range 450–490 nm. The fluorescence intensity images of 1024x1024 pixels and 212x212 µm in size were obtained for visual assessment of the sample structure. FLIM images of 512x512 pixels were acquired from the same fields of view. The FLIM analysis was performed using SPCImage software (Becker & Hickl GmbH, Germany) with a biexponential decay model. To maintain a minimum 5000 counts per pixel, the binning parameter was set at 3. The goodness of fit model was assessed by χ^2 value (it should be at 1). The following parameters were analyzed in 20-30 regions of cells cytoplasm for each field of view: tm (ps), the amplitude-weighted mean lifetime; t1 (ps), the fluorescence lifetime of the free form of NAD(P)H (the short decay component); t2 (ps), the fluorescence lifetime of the bound form of NAD(P)H (the long decay component); and the relative contributions of the free, a1 (%), and the bound, a2 (%), forms of NAD(P)H.

Below we have provided Figures showing the bi-exponential fitting and fluorescence decay of NAD(P)H for each animal group of this study.

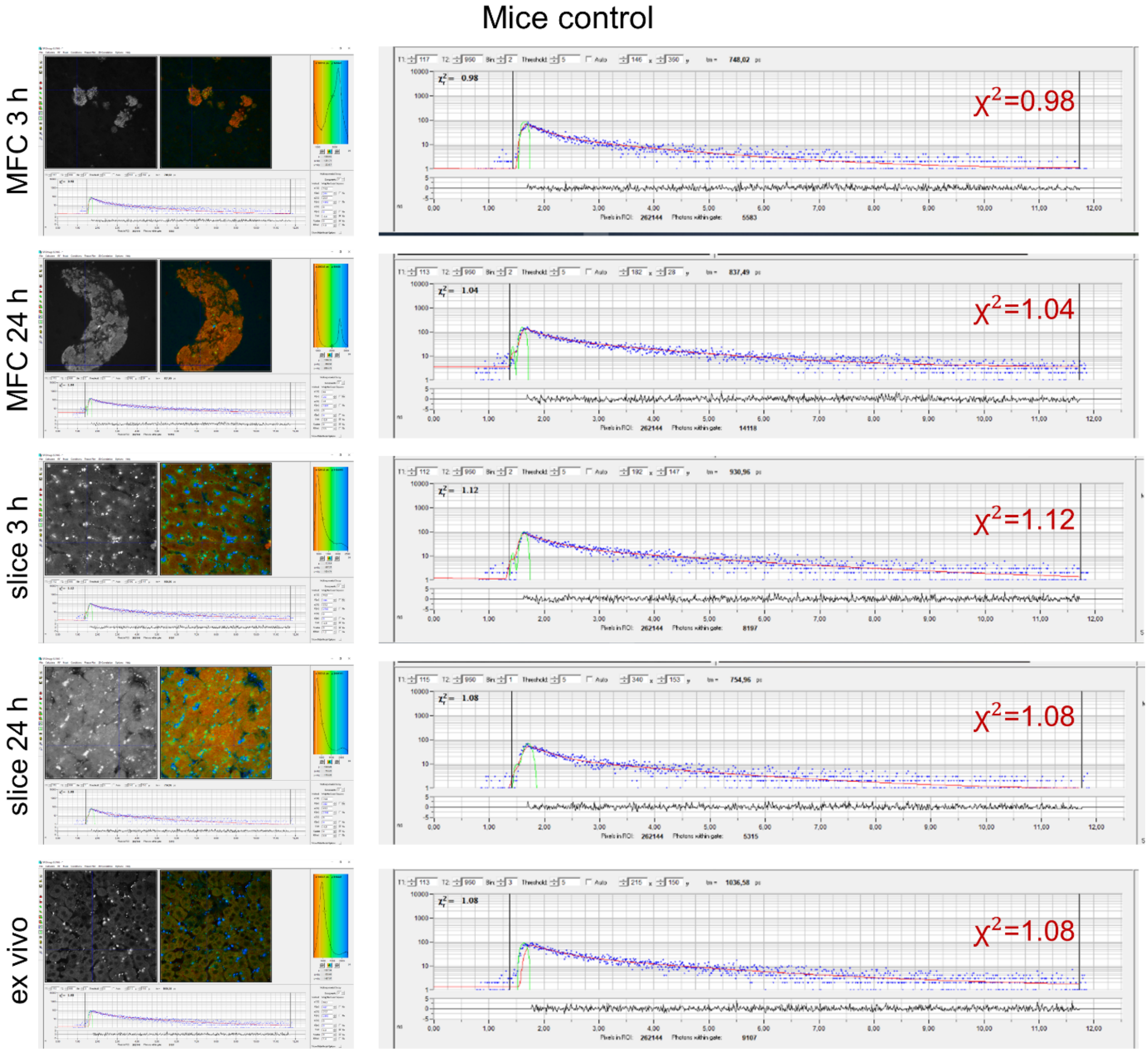

Figure S4. Image of bi-exponential fitting and fluorescence decay of NAD(P)H for the control mice models. The images were obtained using SPCImage software. The values of the goodness of the fit have been marked in red.

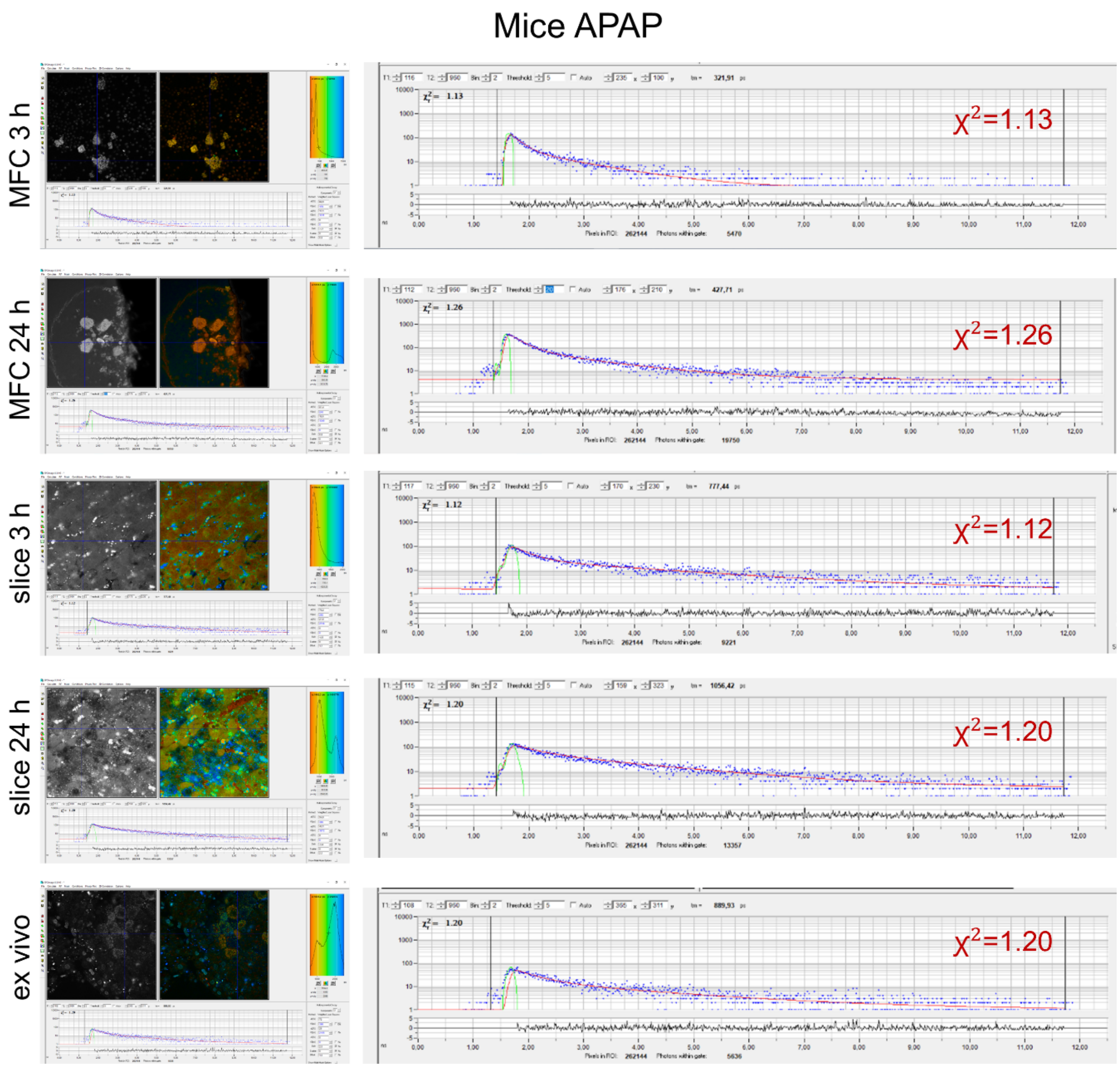

Figure S5. Image of bi-exponential fitting and fluorescence decay of NAD(P)H for the APAP mice models. The images were obtained using SPCImage software. The values of the goodness of the fit have been marked in red.

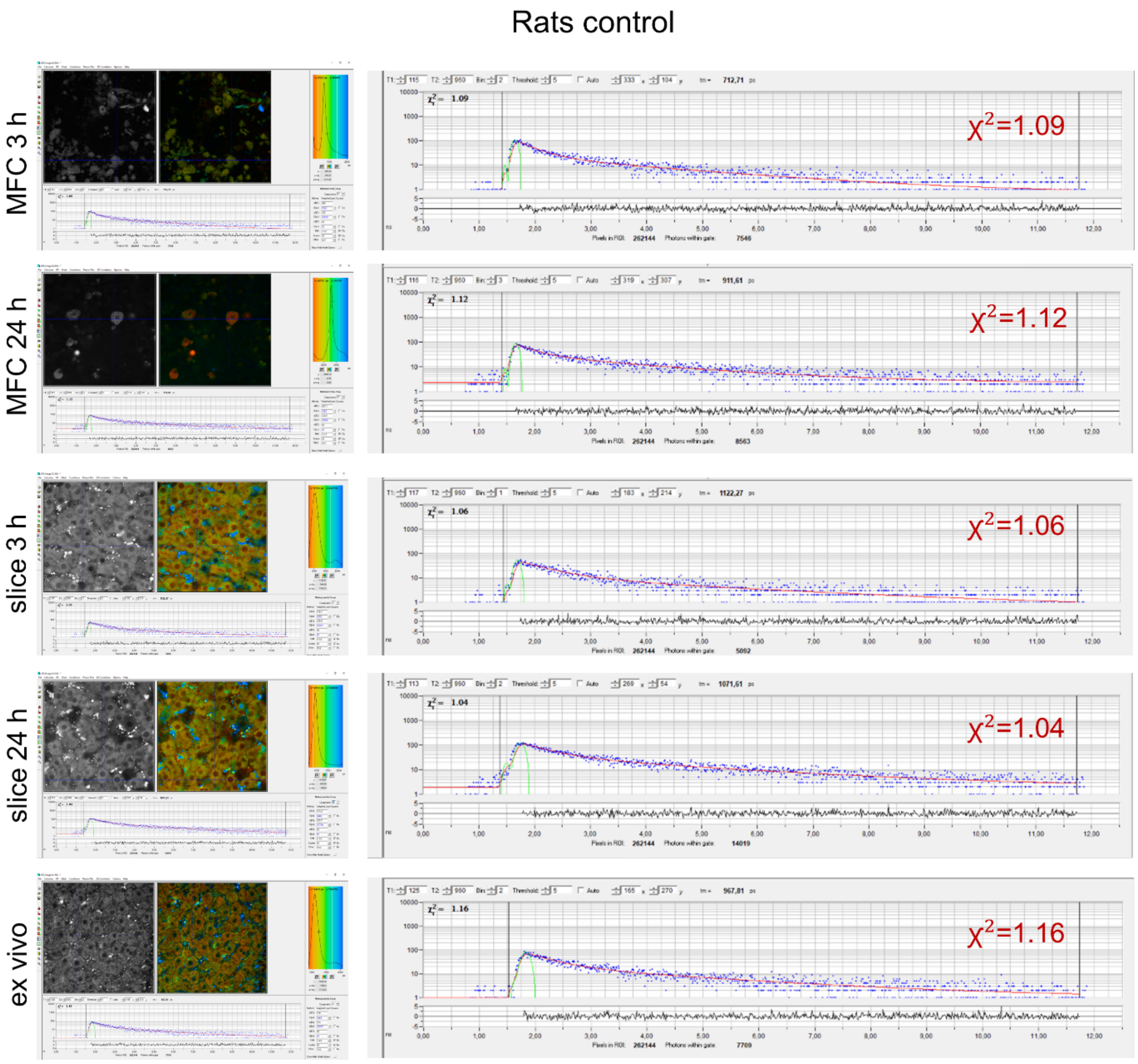

Figure S6. Image of bi-exponential fitting and fluorescence decay of NAD(P)H for the control rat models. The images were obtained using SPCImage software. The values of the goodness of the fit have been marked in red.

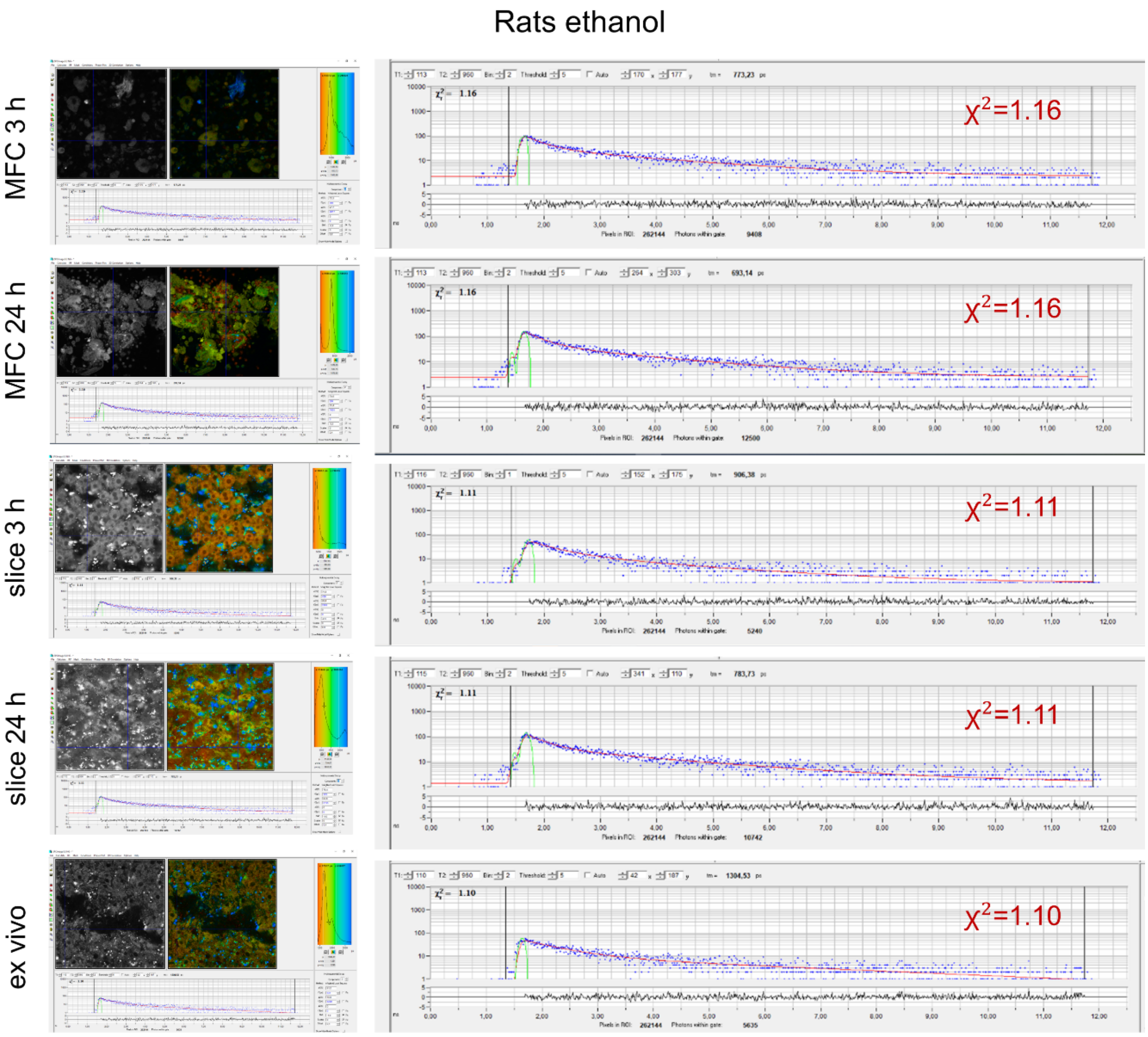

Figure S7. Image of bi-exponential fitting and fluorescence decay of NAD(P)H for the ethanol rat models. The images were obtained using SPCImage software. The values of the goodness of the fit have been marked in red.

Figures S8-S11 show NAD(P)H autofluorescence images (with and without he bright field channel) for all the studied groups, in addition to pseudo-coded FLIM images.

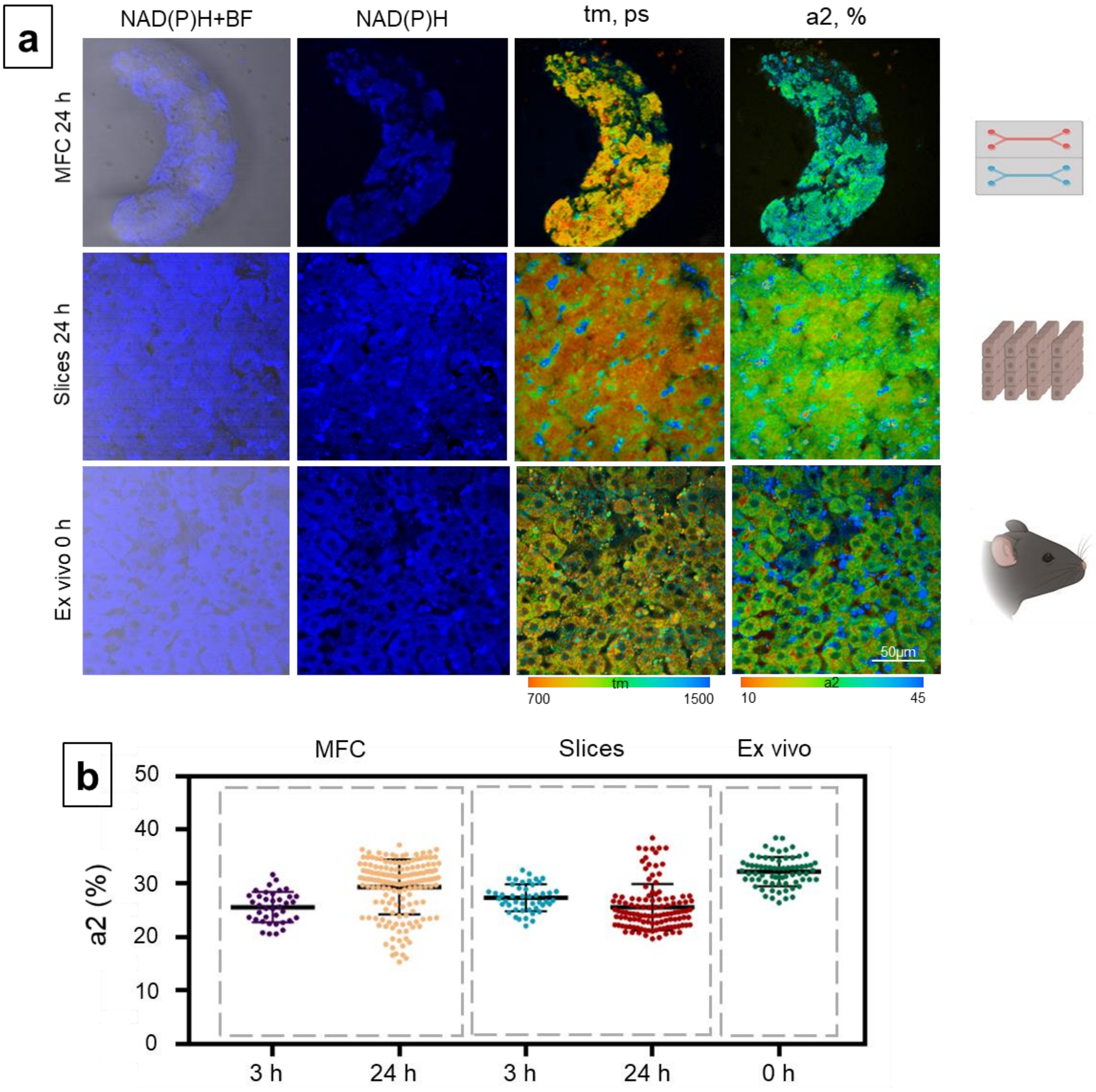

Figure S8. Metabolic imaging of the control mouse hepatocytes for the MFC, slice and ex vivo models. (a) Combined images of the bright-field and autofluorescence of the NAD(P)H, separate autofluorescence of NAD(P)H with pseudo-color-coded fluorescence life-time imaging microscopy (FLIM) images of the NAD(P)H of the hepatocytes, field of view is 213 × 213 μm (512 × 512 pixels); (b) scatter plots reflecting the distribution of the values of the fluorescence lifetime contributions of the bound form of NAD(P)H. Here, tm (ps) is the amplitude-weighted mean lifetime (tm); a2 (%) is the relative contribution of the bound form of NAD(P)H.

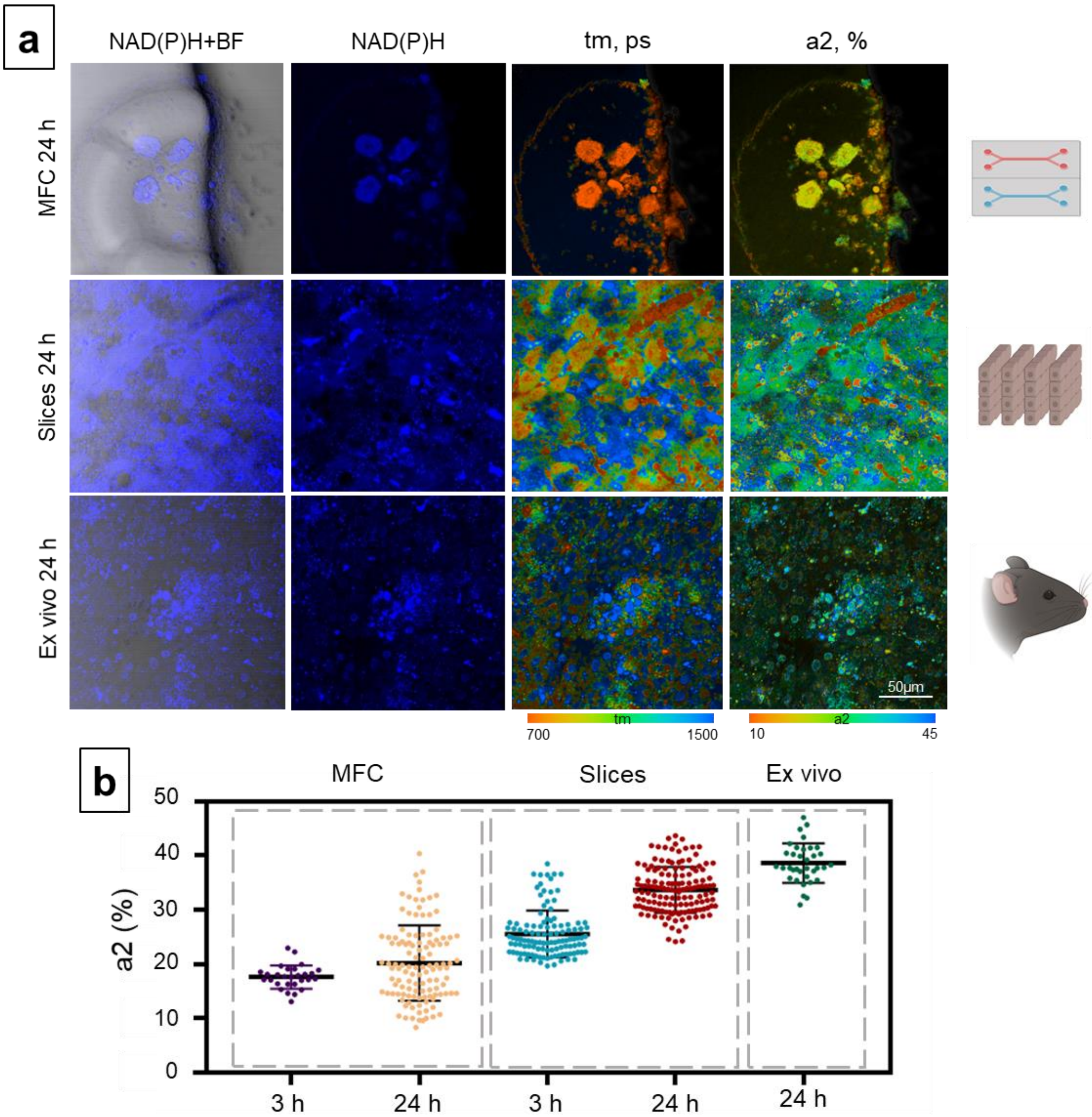

Figure S9. Metabolic imaging of hepatocytes exposed to APAP for the MFC, slice and ex vivo models. (a) Combined images of the bright-field and autofluorescence of the NAD(P)H, separate autofluorescence of the NAD(P)H with pseudocolor-coded FLIM images of the NAD(P)H of the hepatocytes, field of view is 213 × 213 µm (512 × 512 pixels); (b) scatter plots reflecting the distribution of the values of the fluorescence lifetime contributions of the bound form of NAD(P)H. Here, tm (ps) is the amplitude-weighted mean lifetime (tm); a2 (%) is the relative contribution of the bound form of NAD(P)H.

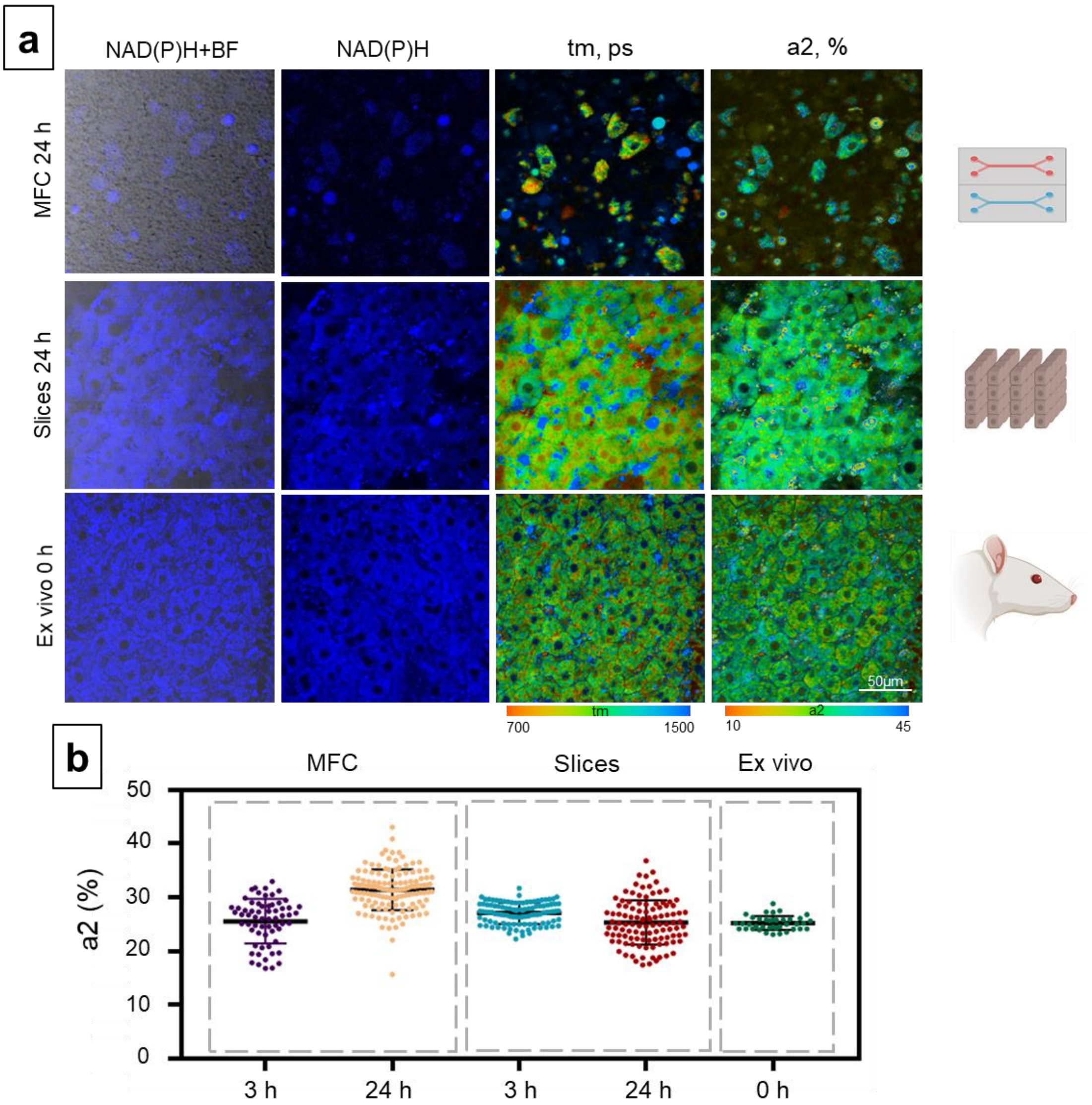

Figure S10. Metabolic imaging of control rats hepatocytes for the MFC, slice and ex vivo models. (a) Combined images of the bright-field and autofluorescence of the NAD(P)H, separate autofluorescence of the NAD(P)H with pseudocolor-coded FLIM images of the NAD(P)H of the hepatocytes, field of view is 213 × 213 µm (512 × 512 pixels); (b) scatter plots reflecting the distribution of the values of the fluorescence lifetime contributions of the bound form of NAD(P)H. Here, tm (ps) is the amplitude-weighted mean lifetime (tm); a2 (%) is the relative contribution of the bound form of NAD(P)H.

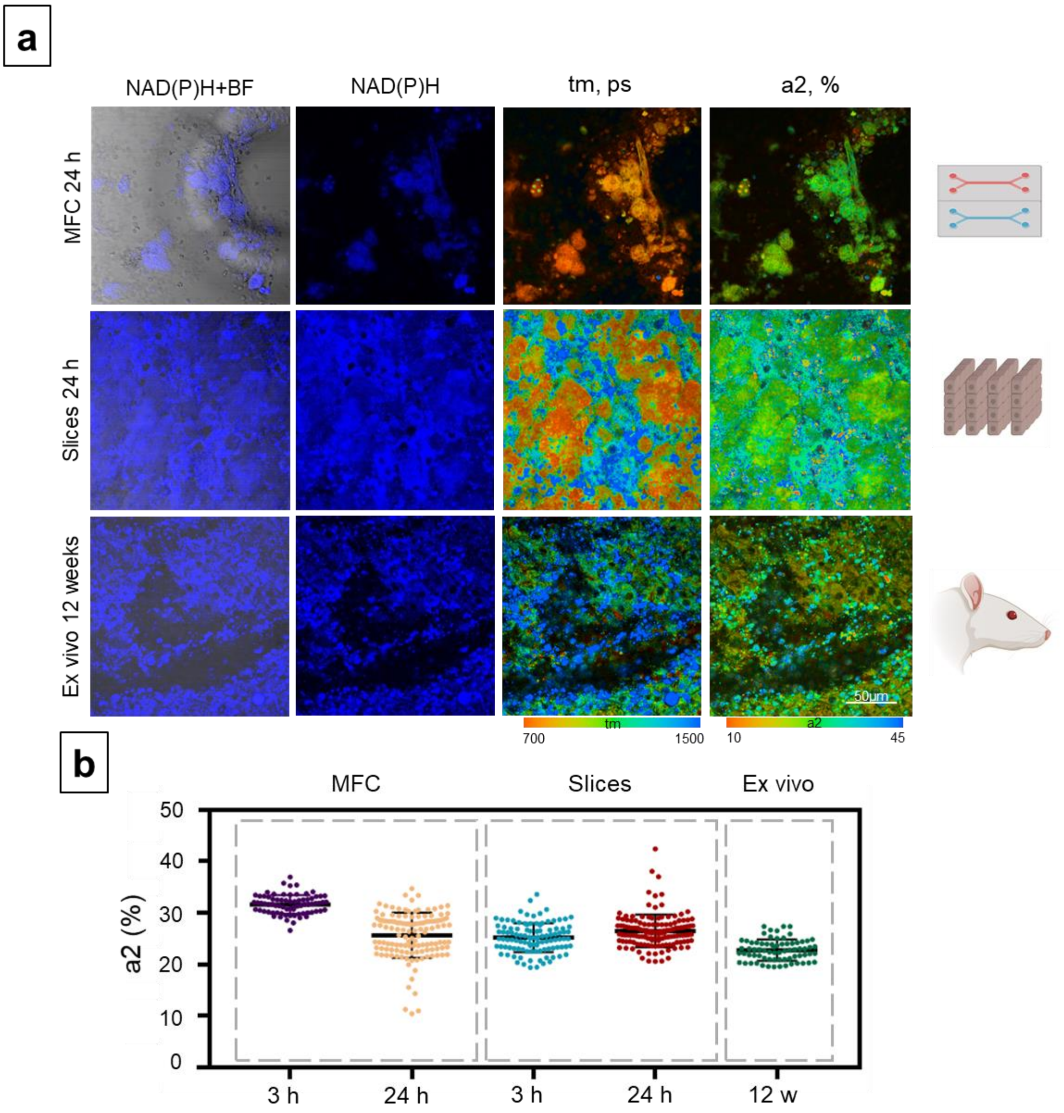

Figure S11. Metabolic imaging of hepatocytes exposed to ethanol for the MFC, slice and ex vivo models. (a) Combined images of the bright-field and autofluorescence of the NAD(P)H, separate autofluorescence of the NAD(P)H with pseudocolor-coded FLIM images of the NAD(P)H of the hepatocytes, field of view is 213 × 213 µm (512 × 512 pixels); (b) scatter plots reflecting the distribution of the values of the fluorescence lifetime contributions of the bound form of NAD(P)H. Here, tm (ps) is the amplitude-weighted mean lifetime (tm); a2 (%) is the relative contribution of the bound form of NAD(P)H.

Based on the data on the fluorescence lifetimes of the free and bound forms of NAD(P)H, it is possible to estimate the change in the metabolic activity of cells, which correlates with the cell viability.

The normal values of t1 and t2 lie in the ranges 300-450 ps and 1500-2200 ps, respectively. Various pathological changes can induce shifts in the values of t1 and t2 (Rueck et al. 2014; Yaseen et al. 2013).

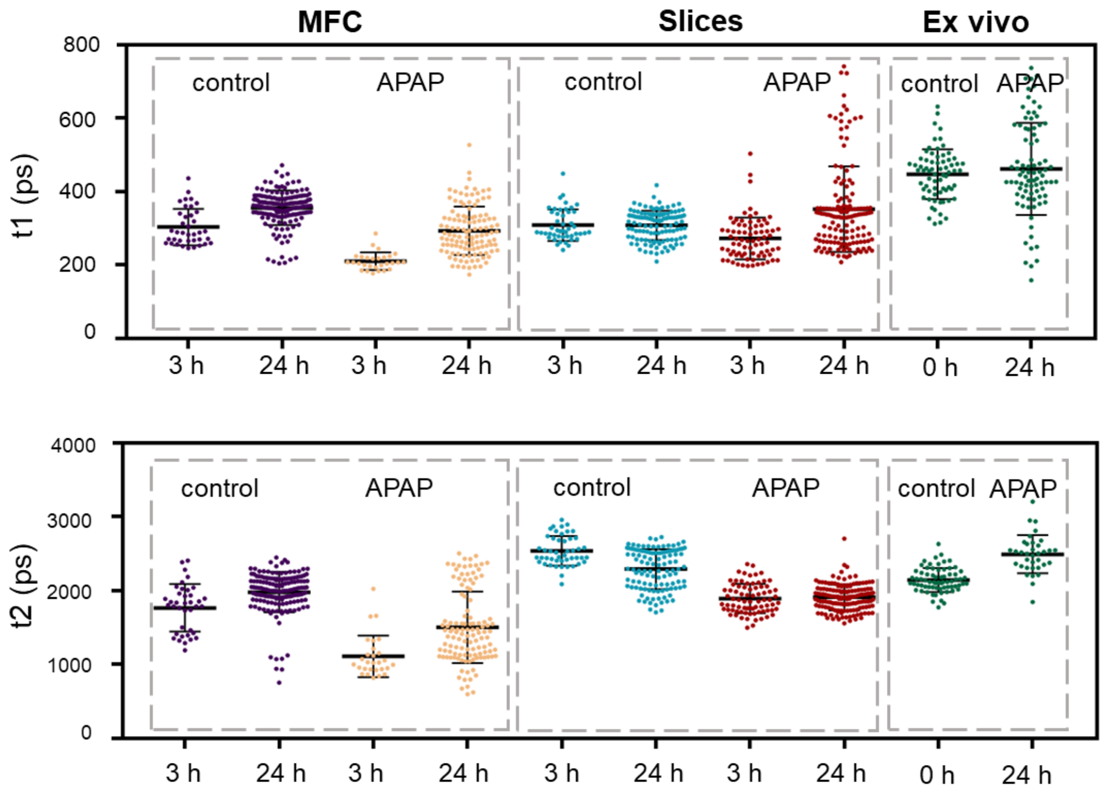

Figure S12. Scatter plots of the fluorescence lifetimes distribution of NAD(P)H for mouse hepatocytes exposed to APAP. t1 (ps) is the fluorescence lifetime of the free form of NAD(P)H, and t2 (ps) is the fluorescence lifetime of the bound form of NAD(P)H

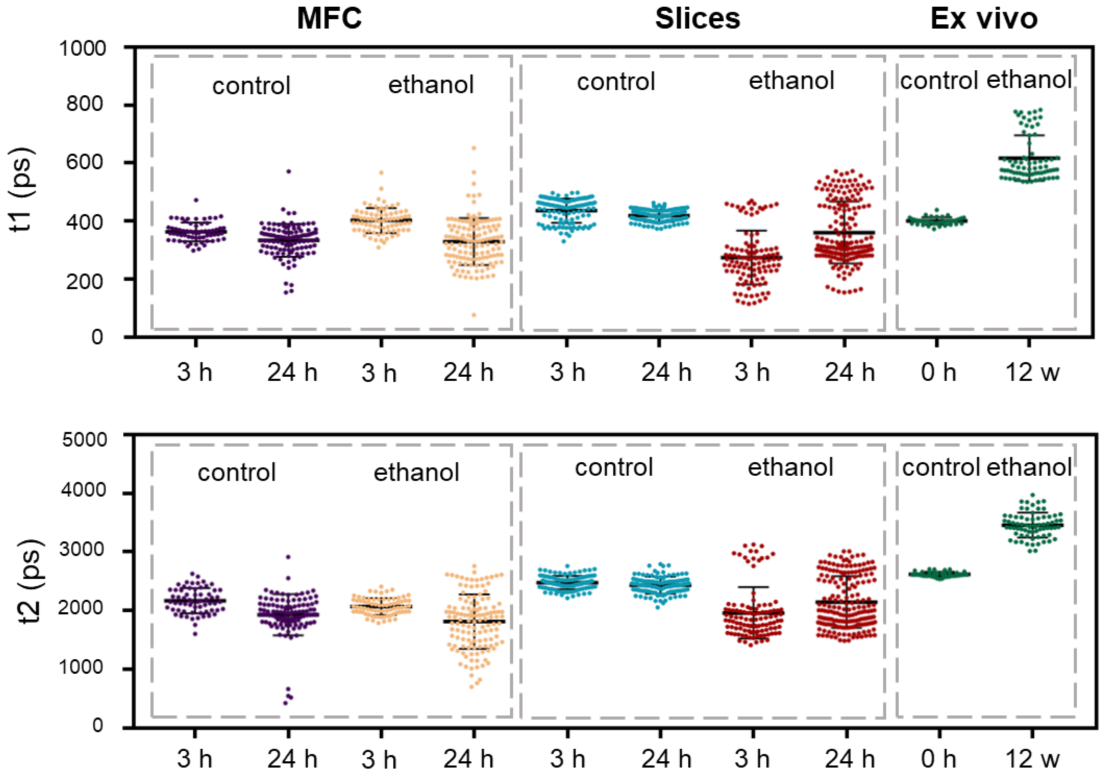

Figure S13. Scatter plots of the fluorescence lifetimes distribution of NAD(P)H for rat hepatocytes exposed to ethanol. t1 (ps) is the fluorescence lifetime of the free form of NAD(P)H, and t2 (ps) is the fluorescence lifetime of the bound form of NAD(P)H

8. Histological analysis

For histological studies, the liver was fixed in a 10% solution of buffered formalin, passed through isopropyl alcohol and embedded in paraffin. Deparaffinized 7 μm sections were stained with hematoxylin and eosin according to the standard protocol (Cardiff et al. 2011). Glass slides that hold the paraffin sections were placed in staining racks. Paraffin from the samples was cleared in three changes of xylene for 2 min per change. To hydrate the samples, the slides were transferred twice through changes of 95% ethanol for 2 min per change, and after that, the slides were transferred to 70% ethanol for 2 min. Next, the slides were rinsed in running tap water at room temperature for at least 2 min. The samples were stained in hematoxylin solution for 3 min and placed under running tap water at room temperature for at least 5 min. After that, the samples were stained in eosin solution for 2 min. To dehydrate the samples, the slides were dipped in 70% ethanol about 20 times. Then, they were transferred to 95% ethanol twice for 2 min. Finally, the samples were washed in three changes of xylene for 2 min per change.

For each sample, ten micrographs were obtained (x40) using a Leica DM 2500 microscope (Germany). Afterwards, a standard morphological analysis of the liver tissue structure was performed, evaluating the presence of dystrophic changes in the cytoplasm of the hepatocytes, such as edema, necrotic cells, fibrosis, and fatty infiltration.

References

1. Kukhtevich IV, Belousov KI, Bukatin AS, Dubina MV, Evstrapov AA. A microfluidic chip with hydrodynamic traps for in vitro microscopic investigations of single cells. Tech Phys Lett. 2015; 41(3): 255-258. https://doi.org/10.1134/S1063785015030086.
2. Guillouzo A. Liver cell models in in vitro toxicology. Environ health persp. 1998; 106(2): 511-532. https://doi.org/10.1289/ehp.98106511.
3. LeCluyse EL, Alexandre E, Hamilton GA, Viollon-Abadie C, Coon DJ, Jolley S, et al. Isolation and culture of primary human hepatocytes. In Basic Cell Culture Protocols. 2005; 207-229.
4. Li WC, Ralphs KL, Tosh D. Isolation and culture of adult mouse hepatocytes. In Mouse Cell Culture. 2010; 185-196. https://doi.org/10.1007/978-1-59745-019-5_13.
5. Jaeschke H, McGill MR, Williams CD, Ramachandran A. Current issues with acetaminophen hepatotoxicity—a clinically relevant model to test the efficacy of natural products. Life sci. 2011; 88(18): 737-745. https://doi.org/10.1016/j.lfs.2011.01.025.
6. Hall PDLM, Lieber CS, DeCarli LM, French SW, Lindros KO, Järveläinen H, et al. Models of alcoholic liver disease in rodents: a critical evaluation. Alcohol Clin Exp Res. 2001; 25: 254S-261S. https://doi.org/10.1111/j.1530-0277.2001.tb02405.x.
7. Palma E, Doornebal EJ, Chokshi S. Precision-cut liver slices: a versatile tool to advance liver research. Hepatol Int. 2019; 13(1): 51-57 https://doi.org/10.1007/s12072-018-9913-7.
8. Van Midwoud PM, Groothuis GM, Merema MT, Verpoorte E. Microfluidic biochip for the perifusion of precision-cut rat liver slices for metabolism and toxicology studies. Biotechnol Bioeng. 2010; 105(1): 184-194. https://doi.org/10.1002/bit.22516.
9. Rueck AC, Hauser C, Mosch S, Kalinina S. Spectrally resolved fluorescence lifetime imaging to investigate cell metabolism in malignant and nonmalignant oral mucosa cells. J Biomed Opt. 2014; 19(9): 096005. https://doi.org/10.1117/1.JBO.19.9.096005.
10. Yaseen MA, Sakadžić S, Wu W, Becker W, Kasischke KA, Boas DA. In vivo imaging of cerebral energy metabolism with two-photon fluorescence lifetime microscopy of NADH. Biomedical optics express. 2013; 4(2): 307-321. https://doi.org/10.1364/BOE.4.000307.
11. Cardiff RD, Miller CH, Munn RJ. Manual hematoxylin and eosin staining of mouse tissue sections. Cold Spring Harbor Protocols. 2014; 2014(6): pdb-prot073411. https://doi.org/10.1101/pdb.prot073411.
